# Parental personality and early life ecology: a prospective cohort study from preconception to postpartum

**DOI:** 10.1038/s41598-023-29139-1

**Published:** 2023-02-27

**Authors:** Elizabeth A. Spry, Craig A. Olsson, Stephanie R. Aarsman, Hanafi Mohamad Husin, Jacqui A. Macdonald, S. Ghazaleh Dashti, Margarita Moreno-Betancur, Primrose Letcher, Ebony J. Biden, Kimberly C. Thomson, Helena McAnally, Christopher J. Greenwood, Melissa Middleton, Delyse M. Hutchinson, John B. Carlin, George C. Patton

**Affiliations:** 1 Centre for Social and Early Emotional Development, School of Psychology, Faculty of Health, Deakin University, Geelong, Australia; 2 Centre for Adolescent Health, Murdoch Children’s Research Institute, Royal Children’s Hospital, Melbourne, Australia; 3 Department of Paediatrics, University of Melbourne, Melbourne, Australia; 4 Clinical Epidemiology and Biostatistics Unit, Murdoch Children’s Research Institute, Royal Children’s Hospital, Melbourne, Australia; 5 Human Early Learning Partnership, School of Population and Public Health, Faculty of Medicine, University of British Columbia, Vancouver, Canada; 6 Centre for Health Evaluation and Outcome Sciences, Providence Health Care Research Institute, Vancouver, Canada; 7 Department of Preventive and Social Medicine, University of Otago, Dunedin, New Zealand; 8 National Drug and Alcohol Research Centre, Faculty of Medicine, University of New South Wales, Sydney, Australia; 9 Centre for Social and Early Emotional Development, Faculty of Health, Deakin University, 221 Burwood Highway, Burwood, VIC 3125 Australia

**Keywords:** Psychology, Human behaviour

## Abstract

Personality reliably predicts life outcomes ranging from social and material resources to mental health and interpersonal capacities. However, little is known about the potential intergenerational impact of parent personality prior to offspring conception on family resources and child development across the first thousand days of life. We analysed data from the Victorian Intergenerational Health Cohort Study (665 parents, 1030 infants; est. 1992), a two-generation study with prospective assessment of preconception background factors in parental adolescence, preconception personality traits in young adulthood (agreeableness, conscientiousness, emotional stability, extraversion, and openness), and multiple parental resources and infant characteristics in pregnancy and after the birth of their child. After adjusting for pre-exposure confounders, both maternal and paternal preconception personality traits were associated with numerous parental resources and attributes in pregnancy and postpartum, as well as with infant biobehavioural characteristics. Effect sizes ranged from small to moderate when considering parent personality traits as continuous exposures, and from small to large when considering personality traits as binary exposures. Young adult personality, well before offspring conception, is associated with the perinatal household social and financial context, parental mental health, parenting style and self-efficacy, and temperamental characteristics of offspring. These are pivotal aspects of early life development that ultimately predict a child’s long-term health and development.

## Introduction

Personality reflects individual patterns of thought, emotions and behaviour, which fundamentally shape the way people engage in social and intimate relationships, education, and work, as well as respond to adversity^1^. Personality traits are often described along five dimensions: agreeableness, conscientiousness, emotional stability/neuroticism, extraversion, and openness^2–4^. These personality traits predict important outcomes ranging from subjective wellbeing and mental health, interpersonal relationships including marriage to occupational success and community engagement. Personality has therefore been described as one of the strongest and most consistent predictors of within-generation consequential and enduring life outcomes^2,5^, with effect sizes equivalent to those of cognitive skills and socio-economic characteristics^6^.

A possibility that these consequences of personality may extend beyond an individual’s own lifespan has received comparatively less attention, despite long-standing theoretical formulations that parent personality plays a central role in shaping child development^7,8^. For example, Belsky’s seminal determinants of parenting model highlights parental personality as a potential determinant of multiple aspects of the social ecology in which children are raised^8^. At one level of this ecology, parents’ personality traits may influence the material and social resources that they bring to the role of parenthood. This idea is consistent with prior evidence that people higher in agreeableness, conscientiousness and emotional stability report increased relationship satisfaction and reduced interpersonal conflict^2,5^, while extraversion predicts size and quality of social supports, and many personality traits predict occupational and financial attainment^2,5^. Such social and material supports are particularly important in the transition to parenthood and children’s early development^9,10^. However, the role of parent personality in shaping this social context into which children are born including social support, intimate partner relationships, life stressors and finances, remains underexplored^11^.

Personality may also influence the individual capacities and interpersonal approach that parents bring to the role of parenting, and the relationship they form with their infant. For example, higher levels of emotional stability, which is broadly associated with reduced risk of life stress and psychopathology^12–14^, may support parents in navigating the unique stressors of pregnancy and early parenthood and manage disruptions to work, relationships, sleep, and daily functioning^15–17^. Prior studies also suggest that on average, parents higher in agreeableness, conscientiousness, emotional stability, extraversion, and openness, engage in warmer and more structured parenting^18^. Thus, through the accumulation and provisioning of relational, and material resources, parents’ preconception personality traits have the potential to shape the perinatal context for offspring.

Parent personality may also play a role in shaping infant biobehavioural characteristics. For example, there is some mixed evidence of associations between maternal personality in pregnancy and offspring preterm birth and birthweight^19^. Earlier studies have also suggested cross-sectional associations between parent personality traits and their infant temperamental correlates^20,21^. For example, maternal neuroticism is correlated with infant stress reactivity. These associations suggest intergenerational inheritance processes which may operate via both biological and environmentally-mediated adaptations. However, given that personality traits change over time^1^, associations between parent personality and infant biobehaviour could reflect bidirectional parent–child effects or influences of perinatal factors on parent reported personality. Examination of parent personality before offspring conception could shed some light on these processes through clearer temporal sequencing, but these prospective associations remain unexplored.

Taken together, emerging evidence suggests potential links between parent personality traits and many aspects of the perinatal ecology including accumulated parental assets and resources, and early-emerging infant biobehavioural characteristics. However, there remain important gaps in knowledge. Much prior research focuses on mothers, or else considers ‘parental’ personality without distinguishing the potentially different roles of mothers and fathers (e.g.^7,15,19,21^). This is in contrast to the broader literature on intergenerational origins of child development, which recognises the potential differences in biological and social pathways for men and women^22,23^. Additionally, prior studies have typically limited the scope of their examination to a single ecological outcome domain (e.g., parent mental health^15–17^, infant birth outcomes^19^, or infant temperament^20,21^). Examining outcomes across multiple domains within the one study can provide a more comprehensive examination of the role of parent personality across the broader perinatal ecology^24^. Lastly, prior research has assessed personality during pregnancy or postpartum, often cross-sectionally with outcomes. Such assessment may be affected by perinatal factors such as sleep disturbance and mental health problems. Moreover, it is now understood that individual differences in personality traits are not fixed but can change over time^1^. Here, our interest is in the role of parents’ personality in the years *before* offspring conception in shaping the perinatal ecology for future offspring^25^.

The purpose of this study was to examine the extent to which parental personality, prior to conception, predicts diverse aspects of the perinatal ecology at the level of the *infant*, the *parent* and broader *social context*. Our overall aim was to provide an initial map of the broad pattern of associations between maternal and paternal preconception personality and diverse perinatal outcomes. Specifically, we examined prospective associations between maternal and paternal pre-pregnancy young adult agreeableness, conscientiousness, extraversion, and openness, and a range of infant, parental, and contextual outcomes during pregnancy and the first year postpartum that are foundational for healthy development. We used data from the Victorian Intergenerational Health Cohort Study (VIHCS), a unique cohort with prospective assessment of women and men up to 10 years before pregnancy, during pregnancy, and postnatally.

## Methods

### Sample

VIHCS is an ongoing prospective intergenerational study of preconception predictors of infant and child health, described elsewhere^26^. It arose from a cohort study commencing in 1992 in the state of Victoria, Australia (The Victorian Adolescent Health Cohort Study; VAHCS)^27^. Briefly, a sample of 1943 Victorian mid-secondary school students (1000 female) were recruited via a two-stage cluster sampling design and assessed six-monthly during adolescence (VAHCS Waves 1–6: mean age 14.9–17.4 years), and three times in young adulthood (VAHCS Waves 7–9: 20.7, 24.1 and 29.1 years). VIHCS began in 2006, during the ninth wave of VAHCS. Between 2006 and 2013 (participants aged 29–36 years), encompassing median maternal and paternal age for Australian births^28^, VAHCS participants were screened six-monthly for pregnancies via SMS, email, and phone calls. Participants reporting a pregnancy or recently born infant were invited to participate in VIHCS, and asked to complete telephone interviews in trimester three, two months postpartum and one year postpartum for each infant born during VIHCS screening. Participants’ parents or guardians provided informed written consent at recruitment into VAHCS, and participants provided informed verbal consent at every subsequent wave. Protocols were developed in accordance with relevant ethical guidelines and research codes, and approved by the human research ethics committee at the Royal Children’s Hospital, Melbourne (VAHCS #21009/33168 and VIHCS #26032).

### Measures

#### Preconception personality traits

Traits are commonly defined across a five-dimensional personality taxonomy, known collectively as the ‘Big Five’: agreeableness, conscientiousness, emotional stability, extraversion, and openness^1,4^. Parents’ pre-pregnancy personality traits were assessed in VAHCS in young adulthood (mean participant age 24.0 years, SD = 1.4, IQR = 23.7–24.4), using the NEO Five Factor Inventory (NEO-FFI)^29^. The NEO-FFI is a 60-item scale that assesses the ‘big five’ domains of personality. It has shown sound reliability, and construct and predictive validity in adult populations including in Australia and Canada^30,31^. Response options ranged from 1 (strongly disagree) to 5 (strongly agree). An overall mean scale score for each personality domain was created, with higher scores reflecting higher levels of the named domain. We then calculated standardised mean scores such that we could assess effects of changes in units of standard deviations. We used these standardised continuous scores for primary analyses. We also created binary personality variables for use in supplementary sensitivity analyses. In the absence of validated cut-points, we dichotomised the continuous score for each trait at ≤ 15th percentile (i.e., ≤ 1 SD below the mean) to denote more extreme low levels of each trait.

#### Perinatal outcomes

A range of indicators of *family material and social resources* (household income, stressful life events, social support, partner relationship quality), *parental capacities and approach* (depressive symptoms, hostile parenting, parental warmth, anxious parenting, parental self-efficacy, parent–infant bonding), and *infant characteristics* (gestational age at birth, birthweight, size for gestational age, temperamental approach, cooperation, distractibility, persistence, reactivity, and rhythmicity) were assessed in trimester three of pregnancy and/or at one year postpartum. All outcomes were assessed by maternal report, except for father mental health which was assessed by paternal report. For each outcome, scale scores were standardised so that effect sizes can be interpreted in units of standard deviations, to facilitate comparison across outcomes and between mothers and fathers. All outcomes were coded such that high scores denote high levels of the named construct. Validated scales were used for most outcomes. Detailed information on these variables, including references for scale development, validity and reliability where available, are provided in Supplementary Appendices A and C.

#### Covariates

As recommended for analysis of multiple outcomes, we adjusted for a common set of potential confounders to facilitate comparison of the adjusted estimates^24^. These were selected according to the modified disjunctive cause criterion, identified in prior literature as pre-exposure variables (to establish temporal sequencing) that were associated with the exposure and/or outcomes, or a proxy for a potential common cause of exposure and outcomes, but unlikely to act as an instrument (associated with outcome only via exposure)^32^. The following variables were included: participants’ parents’ high school non-completion and separation/divorce; participants’ high school non-completion, positive parental bond with own mother and father, life stressors, daily smoking, binge drinking, mental health problems, underweight, and overweight. All were assessed using validated scales or physical measurements by trained researchers as appropriate. Detailed information is in Supplementary Appendices B and C.

### Statistical analysis

We followed an ‘outcome-wide’ framework designed for analysis of multiple outcomes to address an overarching conceptual question^24^. In addition to allowing a comprehensive single investigation, this approach has been designed to (a) improve efficiency of science through presentation of findings in a single, rather than multiple sequential publications; (b) increase transparency and decrease publication bias by facilitating the reporting of null findings, along with those for which there seems evidence of an association; (c) reduce researcher degrees of freedom in analytic decisions by applying one consistent decision set to the analysis of all outcomes (precluding post-hoc analytic changes on a per-association basis); and (d) facilitate comparison of standardised effect sizes across outcomes^24^.

All analyses were conducted separately for mothers and fathers. The characteristics of parents and infants in the study sample were summarised as mean and standard deviation (SD) for continuous variables and frequency and percent for categorical variables. Each parent personality trait was also summarised as mean and SD, in total and stratified by pre-exposure covariates. Pearson’s correlation coefficients were estimated to describe the correlations amongst preconception personality traits, amongst perinatal outcomes, and between personality traits and outcomes.

To model unadjusted and adjusted associations between each personality trait and perinatal outcome we used linear generalised estimating equations (GEE), with an exchangeable correlation matrix to account for family clustering where more than one pregnancy per participant was included. The distributions of outcomes by exposure were assessed using locally weighted scatterplot smoothing (LOWESS) figures, with approximately linear patterns of association deeming modelling of the mean via regression assuming a linear trend for the exposure appropriate. Models were run first unadjusted and then fully adjusted for all pre-exposure covariates. We present effect sizes from the linear regression analyses as standardised beta coefficients, and uncertainty as 95% confidence intervals (CI).

All analyses included participants who responded at least once in each phase (preconception and perinatal). Among these, there were very low levels of missing data on preconception and postnatal variables (1–2% missing data on each of the five personality exposure variables, 0–6% missing data on each of the 16 postpartum variables; Supplementary Table 1). Due to challenges in detecting pregnancies prospectively, a greater proportion of participants missed the antenatal interview resulting in higher missingness on those six variables (25–45%; though nonetheless comparable with other major cohort studies of a similar timescale, particularly in the context of this study’s high retention rates)^33^. Incomplete data were handled using multiple imputation by chained equations^34–36^. We imputed 55 complete datasets separately for mothers and fathers.

Ethics approvals for this study do not permit the data to be made publicly available, due to limitations of participant consent and concerns regarding potential re-identifiability. Upon request, the dataset subset can be made available to a named individual for the purpose of replication of research findings. Requests to access the dataset can be submitted through our institutional data access protocol: https://lifecourse.melbournechildrens.com/data-access/. Analysis code is publicly available at: https://osf.io/cb4g7/?view_only=6956aa75163749078017ada2e40cafe2. Stata 16 was used for all statistical analyses^37^. Figures were created using R [4.2.0]^38^, RStudio [2.5]^39^, readxl^40^, ggplot2^41^, extrafont^42^, ggthemes^43^, and viridis^44^.

## Results

### Sample characteristics

The sample included 398 women with 609 infants, and 267 men with 421 infants. The flow of participants through VIHCS is presented in Supplementary Fig. 1. Demographics of those screened for, identified as eligible for and participating in VIHCS broadly matched those of the original adolescent cohort (VAHCS)^26^. Basic demographics of the sample and proportion of missing data in each variable are presented in Supplementary Table 1. Around one in three participants came from a household where neither parent had completed high school, and one in five participants had parents who were separated or divorced. Men reported more binge drinking in adolescence and higher rates of high school non-completion, while women reported more adolescent mental health problems. The mean gestational age at birth of infants was 39.2 weeks. Standardised mean scores were comparable between men and women for all traits except agreeableness, which was higher in women, and emotional stability, which was higher in men. For both men and women, mean levels of most personality traits varied across pre-exposure participant demographic, mental health, and behavioural characteristics (Supplementary Table 2).

Correlations (a) amongst parent preconception personality traits, and (b) amongst perinatal outcomes, are shown in Supplementary Tables 3 and 4 respectively. The strongest between-trait correlations were observed for emotional stability, correlated with extraversion and conscientiousness in both men and women, and additionally with agreeableness in women (all 0.31 ≤ r ≤ 0.44). Overall, as expected, correlations between perinatal outcomes were typically larger within versus across domains (e.g., parental outcomes correlated with other parental outcomes; Supplementary Table 4). In women, the largest cross-domain correlations were observed between social resources and parental outcomes (e.g., postnatal social support and parent–infant bond r = 0.28) and, in turn, between parenting and infant traits (e.g., hostile parenting and infant reactivity r = 0.25). In men, the social context and parenting were not assessed, so observed cross-domain correlations were fewer. There were correlations (r ≤ 0.14) between paternal income, depressive symptoms, and infant outcomes.

### Associations between each personality trait and perinatal outcome

**Figure 1:**
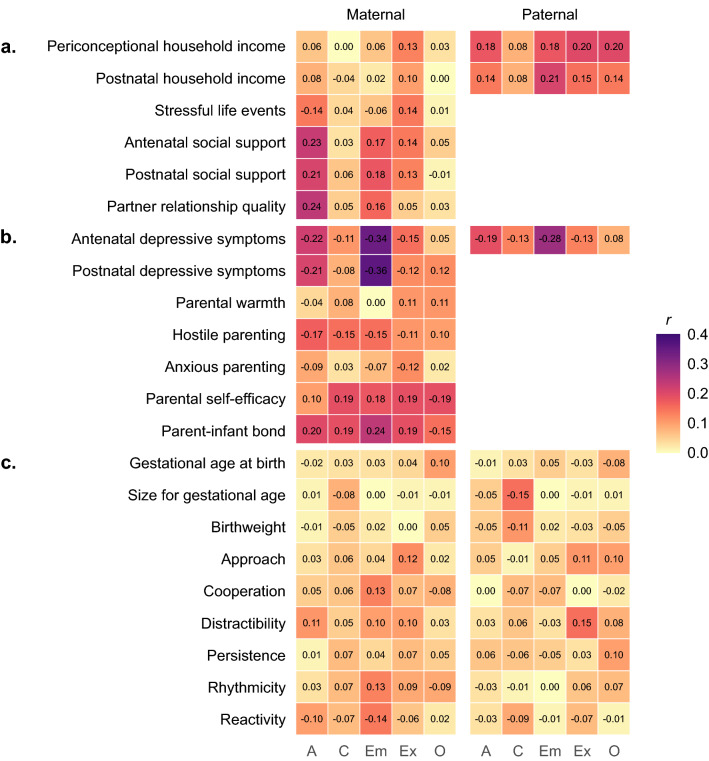
Correlations between parent personality traits and perinatal (**a**) family material and social resources, (**b**) parental capacities and approach, and (**c**) infant biobehavioural characteristics, by sex of parent (maternal n = 609, paternal n = 421), in imputed data. *A* agreeableness, *C* conscientiousness, *Em* emotional stability, *Ex* extraversion, *O* Openness. For all variables, high scores denote high levels of the named construct. Omitted cells indicate that those outcomes were not assessed in fathers. Numbers are Pearson’s r correlation coefficients. Colours represent strength of correlation between parent personality traits (on the horizontal axis) and perinatal outcomes (on the vertical axis), whether positive or negative.

Figure 1 shows the correlations between each preconception personality trait and perinatal outcome, separately for mothers and fathers. Overall, we observed correlations between maternal and paternal personality traits and many perinatal outcomes. For both mothers and fathers, correlations were mostly strongest for family and parental outcomes, and smaller for infant outcomes.

**Figure 2:**
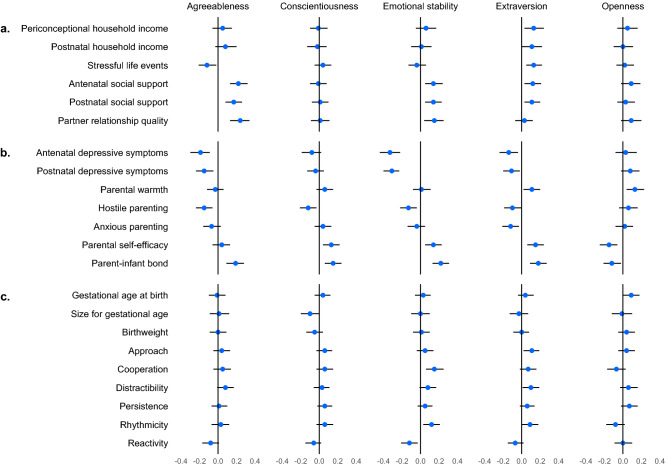
Associations between **maternal** personality traits and perinatal (**a**) family material and social resources, (**b**) parental capacities and approach, and (**c**) infant biobehavioural characteristics (n = 609), in imputed data. Models adjusted for participants’ parents’ high school non-completion and separation/divorce; participants’ high school non-completion, positive parental bond with own mother and father, stressors, daily smoking, binge drinking, mental health problems, underweight, and overweight. For all variables, high scores denote high levels of the named construct. Estimates are presented as standardised betas (β, 95% confidence intervals), interpretable in units of standard deviations.

Figure 2 and Supplementary Table 5 show adjusted associations between each maternal preconception personality trait and perinatal outcome. The observed pattern of correlations remained broadly unchanged after adjustment for all pre-exposure individual, family and demographic confounders. The preconception personality traits for which we observed the strongest adjusted associations with outcomes across domains were emotional stability, extraversion, and agreeableness. Emotional stability was associated with many outcomes across all domains including the social context, parental functioning, and infant traits, with the strongest associations observed for ante- and postnatal depressive symptoms (adjusted antenatal $\widehat{\beta }$ = − 0.33 [95% CI − 0.44, − 0.22]; postnatal $\widehat{\beta }$ = − 0.31 [95% CI − 0.40, − 0.23]). Extraversion was also associated with many outcomes across all domains including the financial and social context, parental functioning, and infant traits, with the strongest associations observed for the parent–infant bond ($\widehat{\beta }$ = 0.18 [95% CI 0.09, 0.27]). Agreeableness was associated with multiple contextual and parental outcomes, with the strongest associations observed for antenatal social support ($\widehat{\beta }$ = 0.22 [95% CI 0.13, 0.32]) and partner relationship ($\widehat{\beta }$ = 0.24 [95% CI 0.13, 0.34]). Conscientiousness and openness were associated with parenting, with the strongest associations observed for conscientiousness with the parent–infant bond ($\widehat{\beta }$ = 0.15 [95% CI 0.06, 0.24]) and openness with parental self-efficacy ($\widehat{\beta }$ = − 0.15 [95% CI − 0.25, − 0.06]).

When considered by outcome domain, of the parental and contextual outcomes, some of the most notable maternal adjusted associations were as follows: household income with extraversion ($$\widehat{\beta }$$ = 0.13, corresponding to a AUD $15,988 [95% CI $3015, $28,962] increase in periconceptional income per standard deviation increase in extraversion); the social context with agreeableness ($$\widehat{\beta }$$ range 0.17–0.24); depressive symptoms with emotional stability, with effect sizes roughly double those of the other maternal traits; and the parent–infant bond with all maternal traits ($$\widehat{\beta }$$ range 0.12–0.22). Of the infant outcomes, approach and distractibility were most strongly associated with maternal extraversion (approach $$\widehat{\beta }$$ = 0.11 [95% CI 0.02, 0.19]; distractibility $$\widehat{\beta }$$ = 0.10 [95% CI 0.01, 0.19]), while cooperation, rhythmicity and reactivity were most strongly associated with maternal emotional stability ($$\widehat{\beta }$$ = range 0.12–0.15).

**Figure 3:**
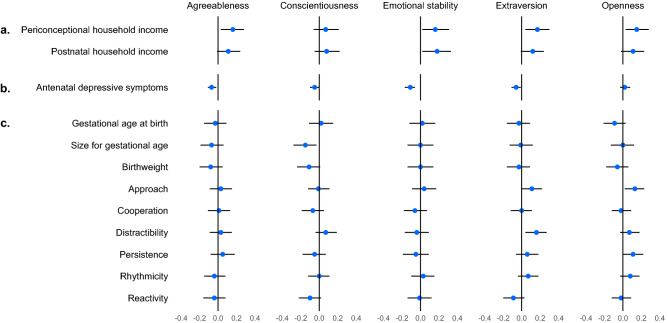
Associations between **paternal** personality traits and perinatal (**a**) family material and social resources, (**b**) parental capacities and approach, and (**c**) infant biobehavioural characteristics (n = 421), in imputed data. Estimates adjusted for participants’ parents’ high school non-completion and separation/divorce; participants’ high school non-completion, positive parental bond with own mother and father, stressors, daily smoking, binge drinking, mental health problems, underweight, and overweight. For all variables, high scores denote high levels of the named construct. Estimates are presented as standardised betas (β, 95% confidence intervals), interpretable in units of standard deviations.

Figure 3 and Supplementary Table 5 show adjusted associations between each paternal preconception personality trait and perinatal outcome. The observed pattern of correlations remained broadly unchanged after adjustment for all pre-exposure individual, family and demographic confounders. For all traits except for conscientiousness, the strongest adjusted associations were observed with perinatal income ($$\widehat{\beta }$$ range 0.11–0.18). For conscientiousness, the strongest association was with infant size for gestational age ($$\widehat{\beta }$$ = − 0.15 [95% CI − 0.28, − 0.03]). When considered by perinatal outcome, the most notable paternal adjusted associations were as follows: household income with all traits, with the strongest associations observed for emotional stability ($$\widehat{\beta }$$ = 0.18, corresponding to a $12,488 [95% CI $1178, $23,799] increase in periconceptional income per standard deviation increase in emotional stability); infant size for gestational age with paternal conscientiousness ($$\widehat{\beta }$$ = − 0.15 [95% CI − 0.28, − 0.03]); and infant approach and distractibility with paternal extraversion (approach $$\widehat{\beta }$$ = 0.11 [95% CI 0.00, 0.22]; distractibility $$\widehat{\beta }$$ = 0.16 [95% CI 0.04, 0.27]) and paternal openness (approach $$\widehat{\beta }$$ = 0.13 [95% CI 0.02, 0.23]).

### Sensitivity analyses

A similar pattern of results was also observed when models were run with binary personality exposures (Supplementary Table 6), though with far larger effect sizes consistent with the more extreme levels of exposure. In women, observed associations were strongest between low agreeableness and each of social support, partner relationship, and maternal infant-bond ($\widehat{\beta }$ ranging from − 0.66 to − 0.82), and between low emotional stability and perinatal depressive symptoms ($\widehat{\beta }$ ranging from 0.59 to 0.62). In men, observed associations were strongest between low emotional stability and income ($\widehat{\beta }$ ranging from − 0.41 to − 0.52). In both men and women, small to moderate associations were also observed between a range of other traits and outcomes, including infant traits, consistent with findings of the continuous exposure analyses.

Restriction of the analysis sample to those with complete data had little effect on the observed associations (Supplementary Table 6). Associations were also mostly robust to mutual adjustment for all other personality traits, with two notable exceptions (Supplementary Table 6). In both men and women, after mutual adjustment for all preconception personality traits, associations with perinatal depressive symptoms largely attenuated for all personality traits except emotional stability and openness. In women, after mutual adjustment for all preconception personality traits, associations with perinatal social support and hostile parenting largely attenuated for all personality traits except agreeableness.

## Discussion

Both maternal and paternal personality traits assessed up to 10 years before pregnancy predict numerous and diverse contextual, parental and infant outcomes in offspring early life. Effect sizes associated with incremental change in personality were small to moderate, but larger when considering a binary exposure of extreme (low) versus normative levels. The pattern of results was robust to adjustment for confounding factors and substantially consistent across sensitivity analyses. These findings highlight a potential role of preconception personality in shaping multiple aspects of the perinatal social ecology^8^. In turn, these elements of the perinatal context have previously been found to predict children’s health and development well into adulthood^45^.

Overall, effect sizes were largest for perinatal family and parental resources and approaches including income, social support, quality of relationships, mental health, and parenting. This matters, because prior evidence suggests powerful impacts of these ecological factors on children’s ongoing development^9,10^. Our observed effect sizes were similar to those reported in prior studies of associations between personality and within-generation outcomes, as well as next-generation parenting of older children^2,5,7,18^. In particular, higher levels of some personality traits—extraversion, emotional stability, and agreeableness—were associated both with reduced parental and contextual risks (e.g., depressive symptoms and stressors) and increased protective factors (e.g., social support, parental self-efficacy). Higher levels of these traits, reflecting interpersonal orientation and emotional regulation capacities, may have some adaptive qualities in pregnancy and parenthood of infants. For example, such traits may facilitate garnering of social and financial capital that in turn support family functioning, by reducing stressors and providing resources and space for parents to focus on attending to infant needs^46^. They may also align with the specific role demands of early parenthood, including a capacity to manage stress and change, empathy that facilitates responsive nurturing, and joy in interacting with infants. These findings align with the suggestion that personality traits are useful, policy-relevant markers of context-dependent health risk and resilience^47,48^, and extend this to an intergenerational setting.

While smaller, the associations between parent personality and infant characteristics were also notable. For example, we observed associations between parent personality traits and their infant temperamental correlates (e.g., parental extraversion with infant orientation-related traits; maternal emotional stability with infant reactivity and regulation-related traits). This finding expands prior work by prospectively demonstrating the early origins of these continuities from before conception and in infancy^21,49^. Such intergenerational continuities could reflect a genetic biological inheritance^50^. Alternatively, evidence from animal and genetically-informed human studies^51,52^ suggests a social pathway from parental genetic influences via parental characteristics, behaviour and resources that shape children’s development (genetic nurture)^51,53^. Consistent with this idea, we found positive associations between parent extraversion and infant approach (reflecting infant comfort in engaging with people and their environment), as well as many parental and contextual attributes that may influence infant approach (e.g., parental mental health, warmth, confidence, and supportive social context)^54^. Maternal emotional stability similarly predicted both lower infant reactivity and relevant context factors (e.g., increased depressive symptoms; lower social support and paternal income). In turn, these early temperament traits have been found to predict children’s life outcomes^55^. Together, findings raise testable hypotheses that intergenerational outcomes of parental personality could operate at least in part via the perinatal social ecology into which children are born, through accumulation of parental health, interpersonal, and social capital, consistent with the concept of intergenerational niche construction^56^.

Notably, both maternal and paternal personality traits are associated with family context and infant outcomes. The role of fathers in early life family context and child development is often neglected^57^. We found that paternal personality traits are associated with many measured aspects of the perinatal social ecology, with effect sizes in many cases equal or greater than those observed for mothers. For example, consistent with findings of prior within-generation studies^12^, similar associations between lower emotional stability and increased depressive symptoms were observed for both mothers and fathers. Associations between parental extraversion and infant orientation-related traits were also similar for mothers and fathers, consistent with prior cross-sectional findings^21^. On the other hand, higher levels of paternal interpersonal and self-regulatory personality traits were associated with sizable increases in perinatal income, with effect sizes much larger than those for mothers. These differences may reflect paid maternal leave structures in Australia at the time of assessment, which could attenuate associations in this life phase that have been previously observed for women more broadly across the lifecourse^2^. Associations with birth outcomes were also larger for fathers, potentially reflecting different biological processes operating periconceptionally^22,23^. In particular, higher paternal conscientiousness was linked to lower infant size for gestational age at birth, potentially reflecting a reduction in larger-for-gestational age babies in fathers with healthy exercise and dietary patterns before offspring conception^58,59^. Our findings thus highlight the important, and potentially distinct contribution of fathers’ personality to early life family context and child development.

### Strengths and limitations

Strengths of this study include the rare longitudinal data, with prospective pre-pregnancy assessment of personality and diverse pre-exposure covariates, and perinatal assessment of outcomes, helping to establish temporal sequencing and reduce the possibility of reverse causation. Our findings were robust to multiple approaches to missing data, exposure definitions, and levels of adjustment. Our multiple outcome approach further circumvents potential for publication bias through nonpublication of ‘null’ findings^24^. Our inclusion of fathers is valuable given the emphasis on maternal influences across the broader perinatal literature.

Our findings should also be considered in light of some limitations. The VAHCS parent cohort was recruited using a stratified random sampling design to be broadly representative of the Victorian adolescent population at the time^27^. Attrition was low in VAHCS, with 85% of participants retained across the 20 year study period and little evidence that those participating in VIHCS differed from the eligible or baseline VAHCS sample^26^. As with all cohort studies, however, the possibility of differential recruitment, attrition and non-response, and changing population demographics over time may have led to underrepresentation of some population groups. Additionally, the study included infants born when parents were aged between 29 and 36 years to maximise recruitment across the period of peak fertility in Australia^28^. Future research should explore generalisability of results to older or younger parents, and across other population groups and settings.

Missing data within the achieved sample was very low for the five exposures, 10 covariates, and 16 postnatal outcomes, but higher for the six antenatal items (though still low compared with major cohorts of a similar timescale)^33^. VIHCS is one of very few long-term preconception cohorts with antenatal data^60^, due in part to the challenges of prospectively identifying all pregnancies to an existing cohort. We used multiple imputation to address potential biases using rich exposure, outcome and covariate information, a robust strategy at higher levels of missing data than here^61^, and note that for constructs assessed both antenatally and postnatally when missing rates were very low, the associations with personality were similar, suggesting minimal bias. We adjusted for a range of pre-exposure confounders and found little change in effect estimates, but potential for residual confounding also remains. In addition, while the present study contributes new evidence on preconception personality in young adulthood, future research may build on these findings by examining the dynamic role of personality across the preconception parental lifecourse.

Data were collected via self- and maternal-report, as is common in longitudinal studies of this timeframe. Parents’ perceptions of child, family, and own psychosocial wellbeing are important in their own right and reliable predictors of next generation outcomes^62,63^. For example, maternally reported infant inhibition and reactivity predict diagnostically assessed psychiatric disorder in childhood and adolescence^63,64^. Prior studies have also found good agreement between maternal report and medical records of birthweight and gestational age at birth^65–67^. Importantly, associations with paternal personality traits were less susceptible to these reporting biases because all outcomes except for paternal depressive symptoms were reported by mothers. Nonetheless, future research should include observational and informant-rated measures to address potential biases due to measurement error.

It should be noted that standardised effect sizes associated with incremental changes in preconception personality were mostly small to moderate. This is unsurprising given the complexity of the perinatal ecology and its many influences. Yet, small effects matter in concert and at scale^68^. Further, small standardised effects can correspond to meaningful real-life differences; for example, in fathers, the observed standardized beta of 0.18 corresponds to an AUD $12,488 increase in income for every standard deviation increase in emotional stability. Moreover, we found large effect sizes when considering a binary exposure of extreme versus normative personality trait levels, with effect sizes similar to those commonly observed for proximal and concurrent parental exposures^69,70^, a striking finding given our more distal assessment of personality up to 10 years preconception.

## Conclusions

The first 1000 days of life are widely considered foundational in brain development, a sensitive phase in which a biological embedding of the social context carries lifelong consequences for cognitive and emotional capabilities, and ultimately health and wellbeing. We found that many elements of the perinatal social context that influence children’s development are associated with parental personality attributes assessed well before offspring conception. We also found evidence of smaller but potentially important continuities in the highest-order parental personality traits and their infant temperamental correlates, suggesting that these intergenerational continuities are evident from very early life. In turn, these elements of the early family ecology accumulate and interact in complex, nuanced ways to influence children’s ongoing development^10^. Thus, the cumulative intergenerational impacts of parent personality may be substantial^2,68^. Our results highlight the potential intergenerational implications of personality, by contributing to many foundational aspects of the social ecology which children are born and raised. These findings lay a groundwork for future hypothesis-driven research to identify whether associations between parental personality and the perinatal ecology reflect causal processes, the social and biological mechanistic pathways underpinning associations, and the extent to which these pathways might influence children’s ongoing development across the life-course.

## Supplementary Information

Supplementary Information.

## Data Availability

Code for analysis from this paper has been made publicly available at: https://osf.io/cb4g7/?view_only=6956aa75163749078017ada2e40cafe2. Ethics approvals for this study do not permit the data to be made publicly available, due to limitations of participant consent and concerns regarding potential re-identifiability. Upon request, the dataset subset can be made available to a named individual for the purpose of replication of research findings. Requests to access the dataset can be submitted through our institutional data access protocol: https://lifecourse.melbournechildrens.com/data-access/.
