# Supplementary material for: Parental personality and early life ecology: a prospective cohort study from preconception to postpartum
Source: Sci Rep. 2023 Feb 27;13:3332. doi: 10.1038/s41598-023-29139-1 (PMC9971123; doi:10.1038/s41598-023-29139-1)
Supplement: Supplementary file 1 — Supplementary Information. [file 41598_2023_29139_MOESM1_ESM.pdf]

Supplementary Information

Parental personality and early life ecology: a prospective cohort study from preconception to postpartum

Elizabeth A. Spry[1,2,3*], Craig A. Olsson[1,2,3], Stephanie R. Aarsman[1,2], Hanafi Mohamad Husin[2], Jacqui A. Macdonald[1,2,3], S. Ghazaleh Dashti[4], Margarita Moreno-Betancur[3,4], Primrose Letcher[1,2,3], Ebony J. Biden[1,2], Kimberly C. Thomson[1, 5,6], Helena McAnally[7], Christopher J. Greenwood[1,2,3], Melissa Middleton [3,4], Delyse M. Hutchinson[1,2,3,8], John B. Carlin[3,4], George C. Patton[2,3]

1. Deakin University, Centre for Social and Early Emotional Development, School of Psychology, Faculty of Health; Geelong, AUSTRALIA
2. Centre for Adolescent Health, Murdoch Children's Research Institute, Royal Children's Hospital, Melbourne, AUSTRALIA
3. University of Melbourne, Department of Paediatrics, Melbourne, AUSTRALIA
4. Clinical Epidemiology and Biostatistics Unit, Murdoch Children's Research Institute, Royal Children's Hospital, Melbourne, AUSTRALIA
5. University of British Columbia, Human Early Learning Partnership, School of Population and Public Health, Faculty of Medicine, Vancouver, CANADA
6. Centre for Health Evaluation and Outcome Sciences, Providence Health Care Research Institute, Vancouver, CANADA
7. Department of Preventive and Social Medicine, University of Otago, Dunedin, NEW ZEALAND
8. University of New South Wales, National Drug and Alcohol Research Centre, Faculty of Medicine, AUSTRALIA

Corresponding author: Dr Elizabeth Spry, Centre for Social and Early Emotional Development, Faculty of Health, Deakin University, 221 Burwood Highway, Burwood, Victoria 3125, Australia. Email: liz.spry@deakin.edu.au

Table of contents

Item	Title	Page
Supplementary Appendix	Outcome and confounder measurement and derivation<br>a. Outcomes<br>b. Covariates<br>c. Reference list	Page 3
Supplementary Table 1	Characteristics of the sample	Page 11
Supplementary Table 2	Mean levels of parent personality traits, by pre-exposure participant characteristics<br>a. Maternal<br>b. Paternal	Page 12
Supplementary Table 3	Correlations between personality traits<br>a. Maternal<br>b. Paternal	Page 14
Supplementary Table 4	Correlations between perinatal outcomes<br>c. Maternal<br>d. Paternal	Page 15
Supplementary Table 5	Table of estimates for Figure 2 and 3:<br>Correlation coefficients and regression estimates<br>a. Agreeableness<br>b. Conscientiousness<br>c. Emotional stability<br>d. Extraversion<br>e. Openness	Page 17
Supplementary Table 6	Sensitivity analyses for associations between parent personality and perinatal outcomes:<br>Complete case, mutually adjusted, binary exposures<br>a. Agreeableness<br>b. Conscientiousness<br>c. Emotional stability<br>d. Extraversion<br>e. Openness	Page 22
Supplementary Figure 1	Study diagram	Page 27

Web Appendix A: Outcome measures

Construct	Description	VIHCS waves assessed	
		Wave 1 (Trimester 3)	Wave 3 (1 year postpartum)
Household income	Household income around immediately prior to conception and after birth were measured using the items "What was your gross household income (in AUD) (pre-tax: including pensions and allowances) that you received in the last financial year from all sources? (before becoming pregnant)?", and "What will be your gross household income (in AUD) (before tax) from all sources for the financial year following the birth of your baby?". Response options ranged from 0 (less than $10,000 p/a) to 18 (more than $200,000 p/a). Each item was converted to a continuous scale by taking the median value of the range provided in each response option. For the last option (more than $200,000 p/a), we replaced the value with $225000.	✓	
Stressful life events	Stressful life events were assessed using the List of Threatening Experiences (LTE) [1]. This scale contains 12 categories of events including illness, death, relationship difficulties, financial difficulties and encounters with crime of the respondent and those around them (e.g., "In the last 12 months, has someone in your family suffered a serious illness, injury or assault"). It has shown sound construct validity and temporal stability in large population-based cohorts [2]. Participants who responded "yes" further reported how much distress that event caused them (responses ranging from 0 (none) to 3 (very much)), and how long the distress lasted in weeks. A total score was created by summing the number of life events where participants reported experiencing "quite a lot" or "very much" distress.	✓	

Construct	Description	VIHCS waves assessed	
		Wave 1 (Trimester 3)	Wave 3 (1 year postpartum)
Social support	The Maternity Social Support Scale (MSSS) [3] used to assess how often the mother feels supported and loved by family, friends and her partner, and how often she has conflict with her partner (e.g., “I have good friends who support me”). This scale contains six items, with response options ranging from 1 (never) to 5 (always). Items 4 and 5 were reversed scored. Participants who were not in a relationship were not asked items pertaining to partner social support (items 3-6). The scale has shown good reliability and predictive utility [3,4]. An overall mean score was derived by calculating the average of responses across items, where higher scores indicated greater social support.	✓	✓
Partner relationship quality	Partner relationship quality was assessed using seven items from the Dyadic Adjustment Scale (DAS) [5], a measure of intimate relationship satisfaction (e.g., “Do you confide in your partner?”) that assesses dyadic satisfaction, dyadic consensus, dyadic cohesion and affectional expression. Response options range from 0 (all the time) to 5 (never). Items 1 and 6 were reverse scored to indicate greater relationship satisfaction, and items were averaged to create an overall mean score.	✓	
Maternal depressive symptoms	Maternal depressive symptoms were assessed using the Edinburgh Postnatal Depression Scale (EPDS) [6]. The EPDS is a 10-item rating scale designed to screen for postpartum depressive symptoms and also validated for antenatal use [7]. Response options range from 0 to 3. Items 1, 2 and 4 were reverse scored such that higher scores indicate more depressive symptoms, and all items were summed to create an overall total score.	✓	✓

Construct	Description	VIHCS waves assessed	
		Wave 1 (Trimester 3)	Wave 3 (1 year postpartum)
Paternal depressive symptoms	Paternal depressive symptoms were assessed using the 12-item General Health Questionnaire (GHQ-12), validated for use in Australian adult populations [8,9] (e.g., "Have you been able to concentrate on what you are doing?"). GHQ-12 examines a person's current level of distress and ability to perform every day functions. Responses ranged from 0 to 4. An overall score was calculated by summing responses, where higher scores reflect poor mental health.	✓	
Hostile parenting, parental warmth, anxious parenting, and parental self-efficacy	Measures of hostile, warm, anxious parenting and parenting self-efficacy were assessed using 16 items from the Longitudinal Study of Australian Children (LSAC) [10–12]. These scales have shown sound psychometric properties (including scale reliability and construct validity) in a large population of Australian parents [12]. These items ask the mother about her behaviour towards her child, with a focus on affection and bonding (e.g., "How often do you hug or hold your child for no particular reason?"). Response options range from 1 to 5. An overall score for each parenting behaviour was derived as an average of responses across items, such that higher scores reflect higher scores for the named behaviour.		✓
Parent-infant bonding	The PBQ is designed to screen for parent-infant bonding problems as perceived by the mother and consists of 25 items representing four factors: 'general impaired bonding' (e.g. I feel happy when my baby smiles or laughs - reversed); 'rejection and anger' (e.g. I feel angry with my baby); 'infant-focused anxiety' (e.g. My baby makes me feel anxious); and, 'risk of abuse' (e.g. I feel like hurting my baby) [13]. Response options range from 0 (always) to 5 (never). The scale has demonstrated good reliability, specificity and predictive value for bonding problems diagnosed through expert consensus using interview and case record data [14,15]. Items were reverse scored such that higher scores indicate greater parental-infant bonding, and were averaged to create an overall mean score.		✓

Construct	Description	VIHCS waves assessed	
		Wave 1 (Trimester 3)	Wave 3 (1 year postpartum)
Infant gestational age at birth and birthweight	Infant birthweight, and gestational age at birth were maternally reported (by VAHCS cohort participant or, for male VAHCS participants, by their partner) either at two months postpartum (86% of participants), at one year postpartum (3%), or during wave 10 of the VAHCS parent cohort surveys (11%; mean child age at assessment 3·8 years, SD = 1.9 years). Prior studies have found good agreement between maternal report and medical records of birthweight and gestational age at birth, particularly when continuous data are collected and when reported in the postpartum or early childhood [16–18].		✓
Infant size for gestational age	Size for gestational age was calculated as birthweight z-scores, relative to the British Growth Reference [19,20]. These population norms provide expected distributions of birth weight according to gestational age and sex of infant. Higher scores indicate large infant size for gestational age.		✓
Infant approach, cooperation, distractibility, persistence, reactivity, and rhythmicity	Infant temperament dimensions were assessed using the 30-item Short Temperament Scale for Toddlers [21] developed through the Australian Temperament Project [22] (e.g., "[Name] is pleasant (smiles, laughs) when first arriving in unfamiliar places"). The scale assesses six dimensions of temperament (approach, cooperation, persistence, rhythmicity, distractibility and reactivity). Response options range from 0 (almost never) to 5 (almost always). Items were reverse coded such that higher scores reflected higher levels of the named temperament factor, and were averaged across responses to create mean scores for each factor.		✓

Web Appendix B. Covariate measures

Construct	Description	VAHCS waves assessed
Participants' parents' high school non-completion	At each wave, participants were asked the highest level of education each of their own parents had achieved (e.g., "What is your FATHER's highest level of education?"). Participants' parents' high school non-completion was defined as neither parent had completed high school.	2-8
Participants' parents' separation/divorce	At each wave, participants were asked to indicate whether their own parents were married, separated/divorced, had passed away or other, and the participant's age when this happened. A dichotomous variable was derived to indicate whether participants' parents had experienced a separation or divorce during adolescence.	2-8
Participants' high school non-completion	Participants were asked "What was the last year of secondary school you completed?". Response options were "Year 8", "Year 9", "Year 10", "Year 11"and "Year 12". Participants were also asked "What is your highest educational qualification?" in VAHCS wave 9. High school non-completion was defined as participants who had less than year 12 education.	7-9
Participants' positive parental bond with own mother and father	The 25-item Parental Bonding Instrument (PBI) [24] was administered in adolescence to assess the bond between adolescent participants and their own parents. The PBI consists of two scales: care and overprotection. Responses ranged from 0 (very unlike my parent) to 3 (very like my parent). Positive parental bond was defined as having both parents display high care and low overprotection [24].	5
Participants' life stressors	Stress was measured using the stress subscale of the Stress and Social Support Inventory [23]. The scale included 6-items that assessed 6 different types of stressors: family, home, school, money, social life and relationships (e.g., "Do you have enough money to spend?"). Response options range from 1 (I'm happy) to 3 (It's giving me problems at the moment). Presence of life stressors was defined as experiencing 2 or more stressors that caused the participant problems at one or more adolescent waves.	2-6

Construct	Description	VAHCS waves assessed
Participants' binge drinking	Alcohol use at each adolescent wave was assessed using a 7-day drinking diary which asked participants the number of standard drinks consumed per drinking day. Binge drinking was defined as ≥5 standard drinks per drinking day at one or more adolescent waves.	2-6
Participants' daily smoking	At each adolescent wave, participants were asked to report the number of cigarettes they smoked during the last week. Daily smoking was defined as smoking 6-7 days per week at one or more adolescent waves.	2-6
Participants' mental health problems	At each adolescent wave, adolescent mental health problems were measured using the Revised Clinical Interview Schedule (CIS-R) [25], a structured psychiatric interview designed to assess symptoms of anxiety and depression in community samples. The CIS-R has been validated for use with adolescent populations [26]. At each wave, total score was dichotomised at ≥12 to identify mixed depression-anxiety symptoms at a level lower than major depressive or anxiety disorder, but which a general practitioner would view as clinically significant [25]. Presence of mental health problems was defined as a score of ≥12 at one or more adolescent waves.	2-6
Participants' underweight<br><br>Participants' overweight	At every adolescent wave, adolescent height and weight were measured by two research assistants trained at baseline [27]. Participant weight was measured to the nearest .1 kg with participants in minimal school uniform, using portable digital scales. A weight of 1 kg was deducted from the measure to account for the weight of the residual clothing. Height was measured with shoes removed using a rigid stadiometer and recorded to the nearest centimetre. Self-reported weights and heights were used for those who had left school in the later teenage waves. Underweight was defined as BMI <18.5 at one or more adolescent waves, and overweight was defined as BMI ≥25 at one or more adolescent waves [28].	2-6

Web Appendix C. Reference list for Supplementary Tables 1 and 2

1. Brugha, T. S. & Cragg, D. The list of threatening experiences: the reliability and validity of a brief life events questionnaire. *Acta Psychiatr. Scand.* **82**, 77–81 (1990).
2. Rosmalen, J. G. M., Bos, E. H. & De Jonge, P. Validation of the long-term difficulties inventory (LDI) and the list of threatening experiences (LTE) as measures of stress in epidemiological population-based cohort studies. *Psychol. Med.* **42**, 2599–2608 (2012).
3. Webster, J. *et al.* Measuring social support in pregnancy: Can it be simple and meaningful? *Birth-Issues Perinat. Care* **27**, 97–101 (2000).
4. Webster, J., Pritchard, M. A., Creedy, D. & East, C. A simplified predictive index for the detection of women at risk for postnatal depression. *Birth* **30**, 101–108 (2003).
5. Spanier, G. B. Measuring dyadic adjustment: New scales for assessing the quality of marriage and similar dyads. *J. Marriage Fam.* 15–28 (1976) doi:10.2307/350547.
6. Cox, J. L., Holden, J. M. & Sagovsky, R. Detection of postnatal depression: Development of the 10-item Edinburgh Postnatal Depression Scale. *Br. J. Psychiatry* **150**, 782–786 (1987).
7. Murray, D. & Cox, J. L. Screening for depression during pregnancy with the edinburgh depression scale (EDDS). *J. Reprod. Infant Psychol.* **8**, 99–107 (1990).
8. Goldberg, D. P. *et al.* The validity of two versions of the GHQ in the WHO study of mental illness in general health care. *Psychol. Med.* **27**, 191–197 (1997).
9. Donath, S. The validity of the 12-item General Health Questionnaire in Australia: a comparison between three scoring methods. *Australas. Psychiatry* **35**, 231–235 (2001).
10. Paterson, G. & Sanson, A. %J S. D. The association of behavioural adjustment to temperament, parenting and family characteristics among 5-year-old children. **8**, 293–309 (1999).
11. US Department of Education. *Early Childhood Longitudinal Study, birth cohor*. (2001).
12. Zubrick, S. R., Lucas, N., Westrupp, E. M. & Nicholson, J. M. Parenting measures in the Longitudinal Study of Australian Children: Construct validity and measurement quality, Waves 1 to 4. *LSAC Tech. Pap.* **12**, 1–110 (2014).
13. Brockington, I. *et al.* A screening questionnaire for mother-infant bonding disorders. *Arch. Womens. Ment. Health* **3**, 133–140 (2001).
14. Mathews, T. L., Emerson, M. R., Moore, T. A., Fial, A. & Hanna, K. M. Systematic Review: Feasibility, Reliability, and Validity of Maternal/Caregiver Attachment and Bonding Screening Tools for Clinical Use. *J Pediatr Heal. Care* **33**, 663–674 (2019).
15. Brockington, I. Postpartum psychiatric disorders. *Lancet (London, England)* **363**, 303–310 (2004).
16. Olson, J. E., Shu, X. O., Ross, J. A., Pendergrass, T. & Robison, L. L. Medical record validation of maternally reported birth characteristics and pregnancy-related events: a report from the Children’s Cancer Group. *Am. J. Epidemiol.* **145**, 58–67 (1997).
17. Gartland, D., Lansakara, N., Flood, M., Brown, S. J. %J A. journal of obstetrics & gynecology. Assessing obstetric risk factors for maternal morbidity: congruity between medical records and mothers’ reports of obstetric exposures. **206**, 152. e1-152. e10 (2012).
18. Shenkin, S. D. *et al.* Validity of recalled v. recorded birth weight: a systematic review and meta-analysis. *J Dev Orig Heal. Dis* **8**, 137–148 (2017).
19. Cole, T. J., Freeman, J. V & Preece, M. A. J. British 1990 growth reference centiles for weight, height, body mass index and head circumference fitted by maximum penalized likelihood. *Stat. Med.* **17**, 407–429 (1998).
20. Wright, C. M. *et al.* Growth reference charts for use in the United Kingdom. *Arch. Dis. Child.* **86**, 11–14 (2002).
21. Fullard, W., McDevitt, S. C. & Carey, W. B. Assessing temperament in one- to three-year-old children. *J. Pediatr. Psychol.* **9**, 205–217 (1984).
22. Prior, M., Sanson, A. V & Oberklaid, F. The Australian temperament project. in *Temperament in childhood* (eds. Kohnstamm, G. A., Bates, J. E. & Rothbart, M. K.) 537–554 (John Wiley & Son, 1989).
23. Jenkins, R., Mann, A. H. & Belsey, E. The background, design and use of a short interview to assess social stress and support in research and clinical settings. *Soc. Sci. Med. Part E-Medical*

Psychol. **15**, 195–203 (1981).
24. Parker, G., Tupling, H. & Brown, L. B. A Parental Bonding Instrument. *Br. J. Med. Psychol.* **52**, 1–10 (1979).
25. Lewis, G., Pelosi, A. J., Araya, R. & Dunn, G. Measuring psychiatric disorder in the community: a standardized assessment for use by lay interviewers. *Psychol. Med.* **22**, 465–486 (1992).
26. Patton, G. C. *et al.* A computerised screening instrument for adolescent depression: population-based validation and application. *Soc. Psychiatry Psychiatr. Epidemiol.* **34**, 166–172 (1999).
27. Patton, G. C. *et al.* Overweight and obesity between adolescence and young adulthood: a 10-year prospective cohort study. *J Adolesc Heal.* **48**, 275–280 (2011).
28. Cole, T. J., Flegal, K. M., Nicholls, D. & Jackson, A. A. Body mass index cut offs to define thinness in children and adolescents: International survey. *Br. Med. J.* **335**, 194–197 (2007).

Supplementary Table 1. Preconception and perinatal characteristics of the participants (maternal *n* = 609 infants to 398 women; paternal *n* = 421 infants to 267 men)

	**Paternal**			**Maternal**		
	n	(%)	% missing	n	(%)	% missing
Adolescent characteristics						
Participants' parents' high school non-completion	115	28%	1%	222	37%	1%
Participants' parents' separation/divorce	71	17%	0%	110	18%	0%
High-school non-completion	43	10%	0%	15	2%	0%
Life stressors	72	17%	0%	151	25%	0%
Positive parental bond with own parents	152	42%	15%	194	36%	10%
Binge drinking	213	51%	0%	191	31%	0%
Daily smoking	75	18%	0%	121	20%	0%
Mental health problems	106	25%	0%	290	48%	0%
Underweight or very underweight	43	10%	1%	94	16%	1%
Overweight or very overweight	85	20%	1%	168	28%	1%
	mean	sd	% missing	n	sd	% missing
Young adult personality traits						
Agreeableness	3.6	0.4	1%	3.8	0.4	1%
Conscientiousness	3.8	0.5	1%	3.8	0.5	1%
Extraversion	3.6	0.4	1%	3.6	0.5	1%
Emotional Stability	3.6	0.6	2%	3.3	0.7	2%
Openness	3.2	0.5	1%	3.3	0.5	1%
Perinatal outcomes						
Family material and social resources						
Periconceptional household income	$ 118,179	$ 52,148	45%	$ 117,846	$ 53,939	39%
Postnatal household income	$ 95,306	$ 43,989	44%	$ 93,798	$ 46,336	40%
Stressful life events	-	-	-	0.5	0.8	32%
Antenatal social support	-	-	-	4.6	0.4	32%
Postnatal social support	-	-	-	4.4	0.6	5%
Partner relationship quality	-	-	-	4.3	0.4	33%
Parental capacities and approach						
Antenatal depressive symptoms	0.8	1.5	25%	4.7	3.6	31%
Postnatal depressive symptoms	-	-	-	4.2	3.8	6%
Parental warmth	-	-	-	4.4	0.5	6%
Hostile parenting	-	-	-	1.8	0.5	6%
Anxious parenting	-	-	-	3.1	0.7	6%
Parental self-efficacy	-	-	-	4.3	0.5	6%
Parent-infant bond	-	-	-	4.6	0.3	5%
Infant biobehavioural characteristics						
Gestational age at birth (weeks)	39.2	2.0	17%	39.2	1.8	0%
Birthweight (kg)	3.4	0.6	17%	3.4	0.6	1%
Size for gestational age (z-score)	0.2	1.0	18%	0.3	1.1	2%
Approach	3.2	0.9	5%	3.1	0.9	5%
Cooperation	2.1	0.9	6%	2.1	0.9	5%
Distractibility	3.2	0.7	6%	3.3	0.7	6%
Persistence	2.1	1.0	5%	2.1	0.9	5%
Rhythmicity	2.4	0.6	5%	2.5	0.7	6%
Reactivity	3.6	0.8	5%	3.6	0.8	5%

Note: % is calculated based on those with observed data. Mean units are unstandardised units / scale scores unless otherwise indicated. Omitted cells indicate outcomes not assessed in fathers. Details regarding variable derivation can be found in Supplementary Appendices A and B.

Supplementary Table 2a: Mean levels of maternal personality traits, by pre-exposure participant characteristics

Participant characteristic	Agreeableness M (sd)	Conscientiousness M (sd)	Emotional stability M (sd)	Extraversion M (sd)	Openness M (sd)
Participants' parents' high school non-completion					
Parent/s completed high school	3.78 (0.42)	3.80 (0.53)	3.63 (0.47)	3.34 (0.71)	3.30 (0.48)
Parents did not complete high school	3.77 (0.43)	3.92 (0.46)	3.59 (0.43)	3.25 (0.69)	3.24 (0.47)
Participants' parents' separation/divorce					
Parents are still together	3.80 (0.40)	3.88 (0.47)	3.63 (0.45)	3.33 (0.68)	3.27 (0.47)
Parents separated/divorced	3.69 (0.52)	3.68 (0.62)	3.54 (0.45)	3.18 (0.78)	3.34 (0.50)
High school non-completion					
Participant completed high school	3.78 (0.43)	3.84 (0.51)	3.61 (0.45)	3.31 (0.69)	3.29 (0.48)
Participant did not complete high school	3.71 (0.29)	4.03 (0.38)	3.75 (0.42)	3.09 (0.99)	2.98 (0.41)
Life stressors					
< 2 life stressors	3.82 (0.40)	3.90 (0.47)	3.63 (0.42)	3.37 (0.67)	3.23 (0.45)
>= 2 life stressors	3.66 (0.48)	3.65 (0.56)	3.57 (0.54)	3.09 (0.75)	3.43 (0.52)
Positive parental bond with own parents					
Low parental bond	3.73 (0.43)	3.79 (0.51)	3.60 (0.47)	3.24 (0.72)	3.30 (0.50)
High parental bond	3.87 (0.41)	3.95 (0.49)	3.66 (0.42)	3.52 (0.62)	3.23 (0.47)
Binge drinking					
No binge drinking	3.78 (0.42)	3.83 (0.52)	3.59 (0.46)	3.28 (0.73)	3.30 (0.47)
>= 5 drinks	3.76 (0.42)	3.87 (0.48)	3.68 (0.43)	3.36 (0.65)	3.23 (0.49)
Daily smoking					
No daily smoking	3.80 (0.41)	3.84 (0.50)	3.61 (0.46)	3.31 (0.71)	3.27 (0.48)
Daily smoking	3.67 (0.45)	3.83 (0.55)	3.64 (0.43)	3.28 (0.67)	3.33 (0.48)
Mental health problems					
No mental health problems	3.88 (0.40)	3.91 (0.49)	3.65 (0.43)	3.47 (0.63)	3.22 (0.48)
Mental health problems	3.66 (0.42)	3.77 (0.52)	3.58 (0.47)	3.12 (0.74)	3.34 (0.47)
Underweight					
Not underweight	3.79 (0.43)	3.85 (0.50)	3.63 (0.45)	3.31 (0.69)	3.26 (0.49)
Underweight or very underweight	3.72 (0.42)	3.82 (0.52)	3.52 (0.50)	3.27 (0.74)	3.40 (0.39)
Overweight					
Not overweight	3.79 (0.42)	3.84 (0.49)	3.58 (0.47)	3.35 (0.70)	3.28 (0.48)
Overweight or very overweight	3.74 (0.44)	3.85 (0.54)	3.69 (0.42)	3.20 (0.69)	3.29 (0.47)

Supplementary Table 2b: Mean levels of paternal personality traits, by pre-exposure participant characteristics

Participant characteristic	Agreeableness M (sd)	Conscientiousness M (sd)	Emotional stability M (sd)	Extraversion M (sd)	Openness M (sd)
Participants' parents' high school non-completion					
Parent/s completed high school	3.63 (0.40)	3.77 (0.47)	3.62 (0.45)	3.64 (0.64)	3.29 (0.54)
Parents did not complete high school	3.53 (0.41)	3.86 (0.40)	3.67 (0.37)	3.63 (0.49)	3.11 (0.45)
Participants' parents' separation/divorce					
Parents are still together	3.64 (0.39)	3.80 (0.46)	3.63 (0.42)	3.65 (0.60)	3.24 (0.52)
Parents separated/divorced	3.43 (0.44)	3.75 (0.43)	3.67 (0.45)	3.59 (0.58)	3.27 (0.54)
High school non-completion					
Participant completed high school	3.61 (0.41)	3.79 (0.46)	3.64 (0.43)	3.65 (0.60)	3.28 (0.52)
Participant did not complete high school	3.51 (0.35)	3.86 (0.35)	3.54 (0.37)	3.55 (0.58)	2.92 (0.52)
Life stressors					
< 2 life stressors	3.62 (0.40)	3.82 (0.44)	3.65 (0.43)	3.67 (0.58)	3.24 (0.52)
>= 2 life stressors	3.52 (0.43)	3.65 (0.47)	3.53 (0.40)	3.48 (0.65)	3.31 (0.55)
Positive parental bond with own parents					
Low parental bond	3.63 (0.41)	3.75 (0.47)	3.60 (0.40)	3.62 (0.60)	3.25 (0.53)
High parental bond	3.63 (0.38)	3.90 (0.42)	3.72 (0.43)	3.77 (0.46)	3.24 (0.51)
Binge drinking					
No binge drinking	3.64 (0.41)	3.82 (0.50)	3.57 (0.46)	3.61 (0.67)	3.26 (0.55)
>= 5 drinks	3.56 (0.40)	3.76 (0.40)	3.69 (0.38)	3.67 (0.52)	3.24 (0.50)
Daily smoking					
No daily smoking	3.64 (0.40)	3.80 (0.45)	3.64 (0.42)	3.66 (0.60)	3.25 (0.52)
Daily smoking	3.42 (0.41)	3.75 (0.47)	3.60 (0.44)	3.55 (0.56)	3.23 (0.57)
Mental health problems					
No mental health problems	3.62 (0.39)	3.80 (0.44)	3.64 (0.43)	3.71 (0.56)	3.25 (0.53)
Mental health problems	3.55 (0.45)	3.76 (0.50)	3.60 (0.42)	3.43 (0.66)	3.25 (0.52)
Underweight					
Not underweight	3.61 (0.41)	3.80 (0.43)	3.63 (0.43)	3.65 (0.59)	3.24 (0.51)
Underweight or very underweight	3.56 (0.31)	3.77 (0.64)	3.66 (0.36)	3.53 (0.65)	3.29 (0.64)
Overweight					
Not overweight	3.63 (0.40)	3.79 (0.46)	3.63 (0.44)	3.64 (0.61)	3.27 (0.53)
Overweight or very overweight	3.52 (0.41)	3.82 (0.41)	3.63 (0.39)	3.61 (0.56)	3.16 (0.51)

Supplementary Table 3a. Correlations between <u>maternal</u> personality traits

		1	2	3	4	5
1	Agreeableness	-				
2	Conscientiousness	0.21	-			
3	Extraversion	0.15	0.24	-		
4	Emotional stability	0.35	0.34	0.44	-	
5	Openness	0.03	-0.08	0.01	-0.09	-

Supplementary Table 3b. Correlations between <u>paternal</u> personality traits

		1	2	3	4	5
1	Agreeableness	-				
2	Conscientiousness	0.09	-			
3	Extraversion	0.11	0.28	-		
4	Emotional stability	0.18	0.39	0.31	-	
5	Openness	0.09	-0.06	0.15	0.08	-

Supplementary Table 4a. Correlations between perinatal a. family material and social resources, b. parental capacities and approach, and c. infant biobehavioural characteristics, in mothers

		1	2	3	4	5	6	7	8	9	10	11	12	13	14	15	16	17	18	19	20	21	22
**a.**	1 Periconceptional household income		0.79	-0.02	-0.01	-0.08	-0.06	-0.06	-0.07	-0.01	-0.01	-0.06	-0.07	-0.01	0.02	-0.04	-0.02	0.05	0.09	0.03	0.02	0.00	-0.01
	2 Postnatal household income	0.79	-	0.02	-0.06	-0.11	-0.07	-0.02	-0.06	-0.04	-0.02	-0.13	-0.08	0.01	0.01	0.03	0.02	0.06	0.04	0.03	0.01	0.08	-0.04
	3 Stressful life events	-0.02	0.02	-	-0.15	-0.18	-0.16	0.17	0.18	-0.04	0.06	-0.06	0.02	-0.03	-0.03	0.03	0.01	0.10	0.02	-0.02	0.01	0.01	0.02
	4 Antenatal social support	-0.01	-0.06	-0.15	-	0.63	0.66	-0.34	-0.20	0.08	-0.07	-0.07	0.10	0.13	0.02	-0.04	-0.03	0.09	0.02	0.04	0.08	0.05	-0.09
	5 Postnatal social support	-0.08	-0.11	-0.18	0.63	-	0.53	-0.28	-0.34	0.17	-0.13	-0.07	0.21	0.28	0.01	0.02	0.02	0.07	0.07	0.02	0.13	0.03	-0.08
	6 Partner relationship quality	-0.06	-0.07	-0.16	0.66	0.53	-	-0.31	-0.18	0.03	-0.18	-0.04	0.07	0.16	-0.05	0.03	0.00	0.07	0.02	0.10	0.08	0.07	-0.13
**b.**	7 Antenatal depressive symptoms	-0.06	-0.02	0.17	-0.34	-0.28	-0.31	-	0.42	-0.05	0.13	-0.02	-0.21	-0.22	-0.02	0.02	0.00	-0.03	-0.08	-0.11	-0.02	-0.12	0.10
	8 Postnatal depressive symptoms	-0.07	-0.06	0.18	-0.20	-0.34	-0.18	0.42	-	-0.09	0.28	0.06	-0.24	-0.42	-0.01	0.01	0.01	-0.06	-0.09	-0.07	0.02	-0.13	0.15
	9 Parental warmth	-0.01	-0.04	-0.04	0.08	0.17	0.03	-0.05	-0.09	-	-0.12	0.24	0.22	0.32	0.04	0.00	0.01	0.09	0.02	0.06	0.12	0.03	0.05
	10 Hostile parenting	-0.01	-0.02	0.06	-0.07	-0.13	-0.18	0.13	0.28	-0.12	-	-0.08	-0.29	-0.61	0.03	0.01	0.03	-0.08	-0.22	-0.11	-0.10	-0.09	0.25
	11 Anxious parenting	-0.06	-0.13	-0.06	-0.07	-0.07	-0.04	-0.02	0.06	0.24	-0.08	-	0.01	0.06	-0.02	-0.02	-0.03	-0.12	-0.04	0.04	0.00	-0.03	0.15
	12 Parental self-efficacy	-0.07	-0.08	0.02	0.10	0.21	0.07	-0.21	-0.24	0.22	-0.29	0.01	-	0.47	-0.02	-0.01	-0.02	0.15	0.24	0.03	0.16	0.21	-0.20
	13 Parent-infant bond	-0.01	0.01	-0.03	0.13	0.28	0.16	-0.22	-0.42	0.32	-0.61	0.06	0.47	-	-0.02	0.06	0.03	0.15	0.21	0.06	0.09	0.14	-0.22
**c.**	14 Gestational age at birth	0.02	0.01	-0.03	0.02	0.01	-0.05	-0.02	-0.01	0.04	0.03	-0.02	-0.02	-0.02	-	-0.17	0.51	0.09	0.01	0.02	0.02	0.03	-0.02
	15 Size for gestational age	-0.04	0.03	0.03	-0.04	0.02	0.03	0.02	0.01	0.00	0.01	-0.02	-0.01	0.06	-0.17	-	0.75	0.05	0.05	-0.03	-0.03	-0.03	0.03
	16 Birthweight	-0.02	0.02	0.01	-0.03	0.02	0.00	0.00	0.01	0.01	0.03	-0.03	-0.02	0.03	0.51	0.75	-	0.10	0.04	-0.02	-0.01	-0.01	0.01
	17 Approach	0.05	0.06	0.10	0.09	0.07	0.07	-0.03	-0.06	0.09	-0.08	-0.12	0.15	0.15	0.09	0.05	0.10	-	0.13	0.05	0.10	0.05	-0.09
	18 Cooperation	0.09	0.04	0.02	0.02	0.07	0.02	-0.08	-0.09	0.02	-0.22	-0.04	0.24	0.21	0.01	0.05	0.04	0.13	-	-0.02	0.25	0.11	-0.26
	19 Distractibility	0.03	0.03	-0.02	0.04	0.02	0.10	-0.11	-0.07	0.06	-0.11	0.04	0.03	0.06	0.02	-0.03	-0.02	0.05	-0.02	-	-0.06	0.16	-0.07
	20 Persistence	0.02	0.01	0.01	0.08	0.13	0.08	-0.02	0.02	0.12	-0.10	0.00	0.16	0.09	0.02	-0.03	-0.01	0.10	0.25	-0.06	-	0.02	0.00
	21 Rhythmicity	0.00	0.08	0.01	0.05	0.03	0.07	-0.12	-0.13	0.03	-0.09	-0.03	0.21	0.14	0.03	-0.03	-0.01	0.05	0.11	0.16	0.02	-	-0.18
	22 Reactivity	-0.01	-0.04	0.02	-0.09	-0.08	-0.13	0.10	0.15	0.05	0.25	0.15	-0.20	-0.22	-0.02	0.03	0.01	-0.09	-0.26	-0.07	0.00	-0.18	-

Supplementary Table 4b. Correlations between perinatal a. family material and social resources, b. parental capacities and approach, and c. infant biobehavioural characteristics, in fathers

		1	2	3	4	5	6	7	8	9	10	11	12
**a.**	1 Periconceptional household income	-	0.79	-0.05	-0.12	-0.05	-0.12	-0.01	-0.06	-0.01	-0.06	0.03	-0.07
	2 Postnatal household income	0.79	-	-0.12	0.01	0.00	0.00	0.00	-0.08	-0.05	-0.11	0.10	-0.04
**b.**	3 Antenatal depressive symptoms	-0.05	-0.12	-	-0.14	0.03	-0.07	-0.09	0.06	0.02	0.04	0.01	0.10
**c.**	4 Gestational age at birth	-0.11	0.01	-0.14	-	-0.05	0.61	0.06	0.00	0.10	0.06	-0.07	-0.05
	5 Size for gestational age	-0.06	0.00	0.03	-0.05	-	0.75	0.09	0.03	0.01	-0.02	0.08	0.04
	6 Birthweight	-0.12	0.00	-0.07	0.61	0.75	-	0.13	0.00	0.06	0.02	0.03	0.01
	7 Approach	-0.01	0.00	-0.09	0.06	0.09	0.13	-	0.02	0.08	0.02	0.12	-0.06
	8 Cooperation	-0.07	-0.08	0.06	0.00	0.03	0.00	0.02	-	0.01	0.17	0.06	-0.38
	9 Distractibility	-0.02	-0.05	0.02	0.10	0.01	0.06	0.08	0.01	-	0.02	0.12	-0.04
	10 Persistence	-0.06	-0.11	0.04	0.06	-0.02	0.02	0.02	0.17	0.02	-	0.03	0.07
	11 Rhythmicity	0.04	0.10	0.01	-0.07	0.08	0.03	0.12	0.06	0.12	0.03	-	-0.12
	12 Reactivity	-0.05	-0.04	0.10	-0.05	0.04	0.01	-0.06	-0.38	-0.04	0.07	-0.12	-

Supplementary Table 5a. Associations between parent agreeableness and perinatal outcomes (as shown in Figures 2 and 3).

Outcome	Mothers							Fathers						
		Unadjusted			Adjusted				Unadjusted			Adjusted		
Construct	r	β	95% CI		β	95% CI		r	β	95% CI		β	95% CI	
Periconceptional household income	0.06	0.07	-0.04	0.17	0.05	-0.06	0.15	0.18	0.20	0.08	0.33	0.16	0.03	0.28
Postnatal household income	0.08	0.09	-0.02	0.20	0.08	-0.03	0.20	0.14	0.15	0.02	0.28	0.11	-0.01	0.24
Stressful life events	-0.14	-0.14	-0.23	-0.04	-0.12	-0.21	-0.02							
Antenatal social support	0.23	0.25	0.16	0.34	0.22	0.13	0.32							
Postnatal social support	0.21	0.20	0.11	0.29	0.17	0.08	0.26							
Partner relationship quality	0.24	0.25	0.15	0.36	0.24	0.13	0.34							
Antenatal depressive symptoms	-0.22	-0.23	-0.34	-0.13	-0.19	-0.30	-0.09	-0.19	-0.09	-0.14	-0.04	-0.07	-0.11	-0.02
Postnatal depressive symptoms	-0.21	-0.19	-0.28	-0.10	-0.15	-0.24	-0.05							
Parental warmth	-0.04	-0.04	-0.13	0.05	-0.03	-0.12	0.06							
Hostile parenting	-0.17	-0.17	-0.26	-0.08	-0.15	-0.24	-0.06							
Anxious parenting	-0.09	-0.10	-0.19	-0.01	-0.07	-0.16	0.03							
Parental self-efficacy	0.10	0.08	-0.01	0.18	0.04	-0.06	0.13							
Parent-infant bond	0.20	0.20	0.11	0.29	0.19	0.09	0.28							
Gestational age at birth	-0.02	-0.01	-0.10	0.08	-0.01	-0.10	0.08	-0.01	-0.01	-0.13	0.11	-0.03	-0.15	0.09
Size for gestational age	0.01	0.02	-0.08	0.12	0.01	-0.09	0.12	-0.05	-0.06	-0.19	0.07	-0.07	-0.19	0.06
Birthweight	-0.01	0.00	-0.09	0.09	0.00	-0.09	0.09	-0.05	-0.06	-0.19	0.06	-0.08	-0.20	0.05
Approach	0.03	0.03	-0.06	0.12	0.04	-0.05	0.13	0.05	0.05	-0.06	0.17	0.03	-0.09	0.15
Cooperation	0.05	0.04	-0.05	0.13	0.05	-0.05	0.14	0.00	0.00	-0.12	0.11	0.01	-0.11	0.13
Distractibility	0.11	0.09	0.00	0.18	0.08	-0.01	0.17	0.03	0.04	-0.08	0.16	0.03	-0.09	0.15
Persistence	0.01	0.01	-0.07	0.10	0.01	-0.07	0.10	0.06	0.06	-0.07	0.18	0.05	-0.08	0.18
Rhythmicity	0.03	0.03	-0.07	0.12	0.03	-0.07	0.12	-0.03	-0.01	-0.13	0.10	-0.04	-0.15	0.08
Reactivity	-0.10	-0.09	-0.18	0.00	-0.08	-0.17	0.01	-0.03	-0.05	-0.16	0.07	-0.04	-0.16	0.08

r = Pearson’s correlation coefficient, β = standardised regression coefficient. Adjusted models adjusted for all assessed family of origin, sociodemographic, mental health, and behavioural characteristics.

Supplementary Table 5b. Associations between parent conscientiousness and perinatal outcomes (as shown in Figures 2 and 3).

**Outcome**	**Mothers**							**Fathers**						
		**Unadjusted**			**Adjusted**				**Unadjusted**			**Adjusted**		
**Construct**	**r**	**β**	**95% CI**		**β**	**95% CI**		**r**	**β**	**95% CI**		**β**	**95% CI**	
Periconceptional household income	0.00	-0.01	-0.11	0.09	-0.01	-0.10	0.09	0.08	0.09	-0.05	0.23	0.07	-0.06	0.21
Postnatal household income	-0.04	-0.04	-0.14	0.06	-0.02	-0.13	0.08	0.08	0.10	-0.04	0.23	0.08	-0.05	0.22
Stressful life events	0.04	0.05	-0.04	0.14	0.04	-0.05	0.13							
Antenatal social support	0.03	0.02	-0.07	0.11	-0.01	-0.10	0.08							
Postnatal social support	0.06	0.05	-0.04	0.14	0.01	-0.08	0.10							
Partner relationship quality	0.05	0.05	-0.05	0.15	0.01	-0.09	0.11							
Antenatal depressive symptoms	-0.11	-0.12	-0.21	-0.02	-0.08	-0.19	0.02	-0.13	-0.06	-0.11	0.00	-0.05	-0.10	0.00
Postnatal depressive symptoms	-0.08	-0.07	-0.16	0.02	-0.04	-0.13	0.05							
Parental warmth	0.08	0.07	-0.01	0.16	0.06	-0.03	0.15							
Hostile parenting	-0.15	-0.14	-0.23	-0.05	-0.12	-0.21	-0.03							
Anxious parenting	0.03	0.04	-0.05	0.13	0.03	-0.05	0.13							
Parental self-efficacy	0.19	0.17	0.08	0.26	0.13	0.04	0.22							
Parent-infant bond	0.19	0.19	0.11	0.28	0.15	0.06	0.24							
Gestational age at birth	0.03	0.03	-0.05	0.11	0.04	-0.05	0.12	0.03	0.03	-0.10	0.15	0.02	-0.11	0.15
Size for gestational age	-0.08	-0.08	-0.18	0.01	-0.10	-0.20	0.00	-0.15	-0.17	-0.30	-0.04	-0.15	-0.28	-0.03
Birthweight	-0.05	-0.05	-0.13	0.04	-0.05	-0.14	0.04	-0.11	-0.12	-0.25	0.01	-0.11	-0.24	0.01
Approach	0.06	0.06	-0.03	0.14	0.06	-0.03	0.14	-0.01	-0.01	-0.12	0.11	-0.01	-0.12	0.11
Cooperation	0.06	0.06	-0.03	0.15	0.06	-0.03	0.15	-0.07	-0.08	-0.19	0.04	-0.07	-0.19	0.05
Distractibility	0.05	0.05	-0.03	0.14	0.03	-0.06	0.11	0.06	0.07	-0.05	0.18	0.07	-0.04	0.19
Persistence	0.07	0.06	-0.02	0.14	0.06	-0.02	0.14	-0.06	-0.07	-0.19	0.05	-0.05	-0.18	0.07
Rhythmicity	0.07	0.06	-0.02	0.15	0.06	-0.03	0.15	-0.01	0.00	-0.12	0.11	0.00	-0.12	0.11
Reactivity	-0.07	-0.07	-0.15	0.02	-0.06	-0.15	0.02	-0.09	-0.10	-0.21	0.02	-0.10	-0.22	0.02

r = Pearson's correlation coefficient, β = standardised regression coefficient. Adjusted models adjusted for all assessed family of origin, sociodemographic, mental health, and behavioural characteristics.

Supplementary Table 5c. Associations between parent emotional stability and perinatal outcomes (as shown in Figures 2 and 3).

	Mothers							Fathers						
**Outcome**		Unadjusted			Adjusted				Unadjusted			Adjusted		
**Construct**	r	β	95% CI		β	95% CI		r	β	95% CI		β	95% CI	
Periconceptional household income	0.06	0.07	-0.04	0.17	0.06	-0.05	0.17	0.18	0.21	0.07	0.36	0.16	0.02	0.31
Postnatal household income	0.02	0.02	-0.08	0.13	0.01	-0.10	0.12	0.21	0.24	0.08	0.40	0.18	0.02	0.33
Stressful life events	-0.06	-0.06	-0.15	0.03	-0.04	-0.13	0.06							
Antenatal social support	0.17	0.17	0.08	0.26	0.14	0.05	0.24							
Postnatal social support	0.18	0.17	0.08	0.26	0.14	0.05	0.23							
Partner relationship quality	0.16	0.16	0.06	0.26	0.15	0.04	0.25							
Antenatal depressive symptoms	-0.34	-0.36	-0.45	-0.26	-0.33	-0.44	-0.22	-0.28	-0.13	-0.19	-0.08	-0.11	-0.17	-0.06
Postnatal depressive symptoms	-0.36	-0.34	-0.43	-0.26	-0.31	-0.40	-0.23							
Parental warmth	0.00	0.00	-0.09	0.09	0.01	-0.08	0.11							
Hostile parenting	-0.15	-0.16	-0.25	-0.06	-0.13	-0.22	-0.04							
Anxious parenting	-0.07	-0.08	-0.17	0.01	-0.04	-0.14	0.05							
Parental self-efficacy	0.18	0.18	0.09	0.26	0.14	0.05	0.23							
Parent-infant bond	0.24	0.24	0.15	0.32	0.22	0.13	0.31							
Gestational age at birth	0.03	0.03	-0.06	0.11	0.03	-0.06	0.11	0.05	0.05	-0.09	0.18	0.02	-0.12	0.16
Size for gestational age	0.00	0.00	-0.10	0.09	0.00	-0.10	0.10	0.00	0.00	-0.15	0.14	0.00	-0.14	0.14
Birthweight	0.02	0.01	-0.07	0.10	0.01	-0.08	0.10	0.02	0.01	-0.13	0.15	0.00	-0.14	0.14
Approach	0.04	0.04	-0.05	0.12	0.05	-0.04	0.14	0.05	0.07	-0.06	0.19	0.04	-0.09	0.17
Cooperation	0.13	0.13	0.04	0.22	0.15	0.06	0.25	-0.07	-0.09	-0.21	0.04	-0.06	-0.18	0.07
Distractibility	0.10	0.09	0.00	0.18	0.08	-0.01	0.17	-0.03	-0.03	-0.16	0.09	-0.04	-0.17	0.09
Persistence	0.04	0.03	-0.05	0.12	0.05	-0.03	0.13	-0.05	-0.06	-0.20	0.07	-0.05	-0.19	0.09
Rhythmicity	0.13	0.12	0.03	0.21	0.12	0.03	0.21	0.00	0.03	-0.09	0.16	0.03	-0.10	0.15
Reactivity	-0.14	-0.14	-0.23	-0.06	-0.12	-0.21	-0.03	-0.01	-0.01	-0.14	0.12	-0.01	-0.14	0.12

r = Pearson's correlation coefficient, β = standardised regression coefficient. Adjusted models adjusted for all assessed family of origin, sociodemographic, mental health, and behavioural characteristics.

Supplementary Table 5d. Associations between parent extraversion and perinatal outcomes (as shown in Figures 2 and 3).

Outcome	Mothers							Fathers						
		Unadjusted			Adjusted				Unadjusted			Adjusted		
Construct	r	β	95% CI		β	95% CI		r	β	95% CI		β	95% CI	
Periconceptional household income	0.13	0.13	0.02	0.24	0.13	0.03	0.24	0.20	0.21	0.08	0.34	0.17	0.04	0.30
Postnatal household income	0.10	0.10	-0.01	0.21	0.11	0.00	0.22	0.15	0.17	0.04	0.29	0.12	-0.01	0.24
Stressful life events	0.14	0.14	0.05	0.22	0.13	0.05	0.22							
Antenatal social support	0.14	0.15	0.06	0.24	0.12	0.03	0.21							
Postnatal social support	0.13	0.14	0.05	0.23	0.11	0.03	0.20							
Partner relationship quality	0.05	0.06	-0.04	0.16	0.03	-0.07	0.12							
Antenatal depressive symptoms	-0.15	-0.16	-0.26	-0.06	-0.14	-0.24	-0.04	-0.13	-0.06	-0.12	-0.01	-0.06	-0.11	-0.01
Postnatal depressive symptoms	-0.12	-0.13	-0.22	-0.04	-0.11	-0.20	-0.02							
Parental warmth	0.11	0.12	0.03	0.20	0.11	0.02	0.20							
Hostile parenting	-0.11	-0.11	-0.20	-0.02	-0.10	-0.19	0.00							
Anxious parenting	-0.12	-0.13	-0.22	-0.04	-0.12	-0.21	-0.03							
Parental self-efficacy	0.19	0.18	0.09	0.27	0.15	0.06	0.24							
Parent-infant bond	0.19	0.20	0.11	0.28	0.18	0.09	0.27							
Gestational age at birth	0.04	0.03	-0.05	0.12	0.04	-0.04	0.13	-0.03	-0.02	-0.15	0.10	-0.03	-0.16	0.09
Size for gestational age	-0.01	-0.02	-0.12	0.07	-0.03	-0.13	0.07	-0.01	-0.02	-0.14	0.11	-0.01	-0.13	0.12
Birthweight	0.00	0.00	-0.09	0.08	0.00	-0.09	0.08	-0.03	-0.04	-0.16	0.09	-0.03	-0.16	0.09
Approach	0.12	0.12	0.03	0.20	0.11	0.02	0.19	0.11	0.11	0.00	0.22	0.11	0.00	0.22
Cooperation	0.07	0.07	-0.02	0.16	0.07	-0.02	0.16	0.00	-0.02	-0.13	0.09	0.00	-0.12	0.11
Distractibility	0.10	0.10	0.01	0.19	0.10	0.01	0.19	0.15	0.15	0.04	0.26	0.16	0.04	0.27
Persistence	0.07	0.06	-0.02	0.15	0.06	-0.02	0.14	0.03	0.04	-0.08	0.16	0.06	-0.06	0.18
Rhythmicity	0.09	0.09	0.00	0.18	0.09	0.00	0.18	0.06	0.06	-0.05	0.17	0.07	-0.04	0.18
Reactivity	-0.06	-0.06	-0.15	0.03	-0.07	-0.15	0.02	-0.07	-0.08	-0.19	0.03	-0.09	-0.20	0.03

r = Pearson's correlation coefficient, β = standardised regression coefficient. Adjusted models adjusted for all assessed family of origin, sociodemographic, mental health, and behavioural characteristics.

Supplementary Table 5e. Associations between parent openness and perinatal outcomes (as shown in Figures 2 and 3).

	Mothers							Fathers						
**Outcome**		**Unadjusted**			**Adjusted**				**Unadjusted**			**Adjusted**		
**Construct**	**r**	**β**	**95% CI**		**β**	**95% CI**		**r**	**β**	**95% CI**		**β**	**95% CI**	
Periconceptional household income	0.03	0.04	-0.07	0.16	0.05	-0.06	0.16	0.20	0.17	0.05	0.29	0.15	0.03	0.28
Postnatal household income	0.00	0.01	-0.10	0.12	0.00	-0.10	0.11	0.14	0.13	0.01	0.25	0.11	-0.02	0.23
Stressful life events	0.01	0.01	-0.09	0.11	0.02	-0.07	0.12							
Antenatal social support	0.05	0.06	-0.05	0.16	0.09	-0.02	0.19							
Postnatal social support	-0.01	-0.01	-0.10	0.09	0.03	-0.06	0.13							
Partner relationship quality	0.03	0.04	-0.07	0.15	0.09	-0.02	0.20							
Antenatal depressive symptoms	0.05	0.05	-0.06	0.17	0.03	-0.08	0.15	0.08	0.03	-0.02	0.08	0.02	-0.03	0.08
Postnatal depressive symptoms	0.12	0.11	0.01	0.21	0.08	-0.02	0.18							
Parental warmth	0.11	0.11	0.02	0.20	0.13	0.04	0.23							
Hostile parenting	0.10	0.09	0.00	0.19	0.06	-0.04	0.16							
Anxious parenting	0.02	0.02	-0.08	0.11	0.02	-0.08	0.11							
Parental self-efficacy	-0.19	-0.19	-0.29	-0.10	-0.15	-0.25	-0.06							
Parent-infant bond	-0.15	-0.16	-0.25	-0.06	-0.12	-0.21	-0.02							
Gestational age at birth	0.10	0.09	0.00	0.18	0.09	0.00	0.18	-0.08	-0.08	-0.19	0.04	-0.09	-0.21	0.03
Size for gestational age	-0.01	-0.02	-0.13	0.08	-0.01	-0.12	0.10	0.01	-0.01	-0.13	0.12	0.00	-0.13	0.12
Birthweight	0.05	0.03	-0.06	0.12	0.04	-0.05	0.13	-0.05	-0.05	-0.17	0.06	-0.06	-0.18	0.06
Approach	0.02	0.02	-0.07	0.12	0.04	-0.05	0.13	0.10	0.11	0.00	0.21	0.13	0.02	0.23
Cooperation	-0.08	-0.08	-0.17	0.02	-0.07	-0.17	0.03	-0.02	-0.01	-0.12	0.09	-0.02	-0.12	0.09
Distractibility	0.03	0.03	-0.06	0.12	0.06	-0.03	0.16	0.08	0.08	-0.02	0.18	0.07	-0.03	0.18
Persistence	0.05	0.06	-0.03	0.14	0.07	-0.02	0.16	0.10	0.11	0.00	0.22	0.11	0.00	0.22
Rhythmicity	-0.09	-0.07	-0.17	0.02	-0.08	-0.18	0.02	0.07	0.07	-0.03	0.17	0.08	-0.03	0.18
Reactivity	0.02	0.01	-0.08	0.11	0.00	-0.09	0.10	-0.01	-0.02	-0.12	0.09	-0.02	-0.12	0.09

r = Pearson's correlation coefficient, β = standardised regression coefficient. Adjusted models adjusted for all assessed family of origin, sociodemographic, mental health, and behavioural characteristics.

Supplementary Table 6a. Sensitivity analyses for associations between parent agreeableness and perinatal outcomes.

	Mothers									Fathers								
**Outcome**	**Complete case adjusted**			**Mutual exposure adjustment**			**Binary exposure**			**Complete case adjusted**			**Mutual exposure adjustment**			**Binary exposure**		
**Outcome construct**	**β**	**95% CI**		**β**	**95% CI**		**β**	**95% CI**		**β**	**95% CI**		**β**	**95% CI**		**β**	**95% CI**	
Periconceptional household income	0.08	-0.05	0.20	0.05	-0.06	0.16	-0.16	-0.63	0.31	0.18	0.03	0.34	0.15	0.02	0.27	-0.33	-0.61	-0.04
Postnatal household income	0.11	-0.01	0.24	0.10	-0.02	0.22	-0.19	-0.68	0.29	0.09	-0.06	0.25	0.10	-0.03	0.22	-0.32	-0.60	-0.03
Stressful life events	-0.11	-0.19	-0.02	-0.14	-0.24	-0.04	0.02	-0.44	0.48									
Antenatal social support	0.23	0.13	0.34	0.21	0.12	0.31	-0.79	-1.24	-0.34									
Postnatal social support	0.17	0.07	0.27	0.16	0.07	0.26	-0.82	-1.25	-0.39									
Partner relationship quality	0.13	0.04	0.23	0.22	0.11	0.33	-0.75	-1.25	-0.24									
Antenatal depressive symptoms	-0.20	-0.31	-0.08	-0.13	-0.23	-0.02	0.45	-0.03	0.93	-0.08	-0.13	-0.03	-0.07	-0.12	-0.02	0.13	0.01	0.25
Postnatal depressive symptoms	-0.13	-0.22	-0.04	-0.09	-0.19	0.00	0.46	0.06	0.86									
Parental warmth	-0.02	-0.11	0.08	-0.06	-0.16	0.03	-0.18	-0.57	0.22									
Hostile parenting	-0.15	-0.25	-0.05	-0.13	-0.22	-0.03	0.38	0.01	0.75									
Anxious parenting	-0.11	-0.21	-0.01	-0.10	-0.20	0.00	0.22	-0.16	0.60									
Parental self-efficacy	0.04	-0.06	0.14	0.02	-0.07	0.12	-0.27	-0.63	0.10									
Parent-infant bond	0.18	0.08	0.28	0.13	0.04	0.23	-0.66	-1.01	-0.32									
Gestational age at birth	-0.03	-0.12	0.07	-0.03	-0.13	0.06	-0.14	-0.53	0.24	-0.01	-0.14	0.12	-0.01	-0.13	0.11	0.00	-0.27	0.28
Size for gestational age	0.05	-0.06	0.16	0.04	-0.07	0.14	-0.01	-0.41	0.38	-0.02	-0.15	0.12	-0.06	-0.19	0.07	0.15	-0.15	0.45
Birthweight	0.01	-0.09	0.10	0.00	-0.09	0.10	-0.10	-0.46	0.25	-0.04	-0.18	0.09	-0.06	-0.18	0.07	0.12	-0.17	0.41
Approach	0.06	-0.03	0.16	0.01	-0.08	0.10	-0.05	-0.41	0.31	0.07	-0.06	0.20	0.03	-0.09	0.15	-0.11	-0.38	0.16
Cooperation	0.08	-0.02	0.17	0.00	-0.10	0.10	-0.16	-0.54	0.23	0.00	-0.13	0.12	0.01	-0.11	0.13	0.01	-0.26	0.28
Distractibility	0.08	-0.02	0.17	0.06	-0.03	0.15	-0.20	-0.57	0.16	0.03	-0.10	0.17	0.03	-0.09	0.14	-0.06	-0.32	0.21
Persistence	0.04	-0.05	0.13	-0.01	-0.10	0.08	-0.15	-0.50	0.20	0.05	-0.09	0.19	0.06	-0.07	0.19	-0.13	-0.41	0.15
Rhythmicity	0.02	-0.08	0.12	-0.01	-0.11	0.09	-0.01	-0.39	0.36	-0.06	-0.19	0.07	-0.03	-0.15	0.09	0.06	-0.20	0.33
Reactivity	-0.10	-0.20	-0.01	-0.05	-0.14	0.05	0.13	-0.24	0.49	-0.04	-0.17	0.09	-0.04	-0.16	0.08	0.04	-0.23	0.31

β = standardised regression coefficient. Complete case models included participants with available data on all analysis variables, and were adjusted for all assessed family of origin, sociodemographic, mental health, and behavioural characteristics. Mutual exposure adjustment models were adjusted for all other personality traits. Binary exposure models included the personality trait exposure dichotomised at the 15th %ile, with the risk category representing low levels of the named trait, and were unadjusted.

Supplementary Table 6b. Sensitivity analyses for associations between parent conscientiousness and perinatal outcomes.

	Mothers									Fathers								
Outcome	Complete case adjusted			Mutual exposure adjustment			Binary exposure			Complete case adjusted			Mutual exposure adjustment			Binary exposure		
**Outcome construct**	**β**	**95% CI**		**β**	**95% CI**		**β**	**95% CI**		**β**	**95% CI**		**β**	**95% CI**		**β**	**95% CI**	
Periconceptional household income	-0.01	-0.12	0.11	-0.05	-0.16	0.05	0.01	-0.42	0.43	0.07	-0.09	0.22	-0.01	-0.16	0.14	-0.14	-0.49	0.21
Postnatal household income	-0.02	-0.14	0.09	-0.07	-0.19	0.04	0.12	-0.33	0.57	0.06	-0.09	0.21	0.00	-0.15	0.15	-0.23	-0.58	0.12
Stressful life events	0.06	-0.02	0.14	0.07	-0.02	0.16	-0.14	-0.56	0.27									
Antenatal social support	0.03	-0.07	0.13	-0.07	-0.16	0.03	-0.10	-0.53	0.34									
Postnatal social support	0.01	-0.08	0.11	-0.03	-0.12	0.06	-0.14	-0.54	0.26									
Partner relationship quality	0.04	-0.05	0.13	-0.02	-0.13	0.08	-0.15	-0.62	0.33									
Antenatal depressive symptoms	-0.09	-0.19	0.02	0.02	-0.08	0.12	0.10	-0.38	0.57	-0.04	-0.09	0.01	0.01	-0.05	0.07	0.11	-0.04	0.25
Postnatal depressive symptoms	-0.05	-0.14	0.04	0.06	-0.03	0.15	0.09	-0.30	0.48									
Parental warmth	0.05	-0.05	0.14	0.08	-0.01	0.18	-0.13	-0.50	0.24									
Hostile parenting	-0.13	-0.23	-0.03	-0.08	-0.18	0.02	0.34	-0.01	0.70									
Anxious parenting	0.02	-0.08	0.12	0.10	0.01	0.20	-0.18	-0.54	0.19									
Parental self-efficacy	0.16	0.06	0.26	0.10	0.01	0.19	-0.29	-0.63	0.05									
Parent-infant bond	0.16	0.07	0.26	0.10	0.01	0.19	-0.38	-0.73	-0.04									
Gestational age at birth	0.02	-0.07	0.11	0.03	-0.06	0.12	-0.02	-0.39	0.36	-0.02	-0.15	0.11	0.01	-0.13	0.15	0.04	-0.28	0.36
Size for gestational age	-0.12	-0.23	-0.02	-0.10	-0.20	0.01	0.06	-0.33	0.44	-0.14	-0.27	-0.01	-0.21	-0.35	-0.07	0.23	-0.13	0.58
Birthweight	-0.08	-0.17	0.02	-0.05	-0.14	0.04	0.01	-0.33	0.35	-0.13	-0.27	0.00	-0.15	-0.29	-0.01	0.22	-0.12	0.55
Approach	0.08	-0.02	0.17	0.04	-0.05	0.13	-0.18	-0.51	0.14	-0.02	-0.15	0.10	-0.05	-0.18	0.08	-0.04	-0.33	0.25
Cooperation	0.09	0.00	0.18	0.01	-0.08	0.11	-0.17	-0.53	0.19	-0.07	-0.19	0.06	-0.06	-0.19	0.07	0.07	-0.22	0.36
Distractibility	0.00	-0.09	0.09	0.01	-0.08	0.11	-0.15	-0.50	0.21	0.07	-0.06	0.20	0.07	-0.05	0.20	-0.16	-0.46	0.15
Persistence	0.07	-0.02	0.15	0.05	-0.04	0.14	0.04	-0.29	0.37	-0.03	-0.17	0.11	-0.06	-0.19	0.08	0.13	-0.19	0.44
Rhythmicity	0.08	-0.02	0.17	0.02	-0.07	0.12	-0.15	-0.50	0.19	0.02	-0.10	0.15	-0.02	-0.15	0.11	-0.22	-0.51	0.07
Reactivity	-0.10	-0.20	-0.01	-0.02	-0.11	0.08	0.09	-0.25	0.43	-0.09	-0.21	0.03	-0.10	-0.23	0.03	0.25	-0.05	0.55

β = standardised regression coefficient. Complete case models included participants with available data on all analysis variables, and were adjusted for all assessed family of origin, sociodemographic, mental health, and behavioural characteristics. Mutual exposure adjustment models were adjusted for all other personality traits. Binary exposure models included the personality trait exposure dichotomised at the 15th %ile, with the risk category representing low levels of the named trait, and were unadjusted.

Supplementary Table 6c. Sensitivity analyses for associations between parent emotional stability and perinatal outcomes.

	Mothers									Fathers								
**Outcome**	**Complete case adjusted**			**Mutual exposure adjustment**			**Binary exposure**			**Complete case adjusted**			**Mutual exposure adjustment**			**Binary exposure**		
**Outcome construct**	**β**	**95% CI**		**β**	**95% CI**		**β**	**95% CI**		**β**	**95% CI**		**β**	**95% CI**		**β**	**95% CI**	
Periconceptional household income	0.12	-0.01	0.25	0.02	-0.10	0.14	-0.20	-0.56	0.16	0.16	-0.04	0.36	0.12	-0.04	0.28	-0.41	-0.84	0.02
Postnatal household income	0.02	-0.12	0.15	-0.03	-0.16	0.09	-0.10	-0.46	0.26	0.16	-0.03	0.34	0.18	0.00	0.35	-0.52	-0.96	-0.07
Stressful life events	-0.02	-0.11	0.07	-0.12	-0.23	-0.02	0.01	-0.35	0.37									
Antenatal social support	0.17	0.06	0.28	0.08	-0.03	0.20	-0.22	-0.59	0.15									
Postnatal social support	0.16	0.07	0.26	0.09	-0.01	0.20	-0.20	-0.54	0.14									
Partner relationship quality	0.14	0.05	0.24	0.11	-0.01	0.23	-0.11	-0.48	0.26									
Antenatal depressive symptoms	-0.35	-0.46	-0.23	-0.32	-0.43	-0.20	0.62	0.25	0.99	-0.09	-0.15	-0.03	-0.12	-0.18	-0.05	0.21	0.02	0.41
Postnatal depressive symptoms	-0.30	-0.39	-0.22	-0.33	-0.43	-0.23	0.59	0.29	0.89									
Parental warmth	0.02	-0.07	0.12	-0.05	-0.16	0.05	0.14	-0.16	0.44									
Hostile parenting	-0.16	-0.26	-0.06	-0.06	-0.17	0.05	0.26	-0.03	0.55									
Anxious parenting	-0.04	-0.15	0.06	-0.02	-0.13	0.09	0.19	-0.11	0.49									
Parental self-efficacy	0.19	0.09	0.29	0.06	-0.04	0.17	-0.21	-0.49	0.07									
Parent-infant bond	0.24	0.14	0.34	0.10	0.00	0.20	-0.33	-0.60	-0.05									
Gestational age at birth	0.02	-0.08	0.12	0.03	-0.07	0.13	-0.08	-0.39	0.23	-0.03	-0.19	0.14	0.07	-0.08	0.21	-0.07	-0.48	0.34
Size for gestational age	-0.03	-0.14	0.08	0.02	-0.09	0.14	0.16	-0.15	0.47	0.04	-0.11	0.20	0.09	-0.07	0.25	0.19	-0.25	0.64
Birthweight	-0.01	-0.11	0.09	0.04	-0.06	0.14	0.07	-0.22	0.35	-0.03	-0.20	0.13	0.10	-0.06	0.26	0.10	-0.35	0.54
Approach	0.08	-0.02	0.17	-0.03	-0.13	0.08	-0.07	-0.35	0.20	0.06	-0.09	0.21	0.04	-0.10	0.18	-0.30	-0.70	0.10
Cooperation	0.15	0.06	0.24	0.11	0.00	0.22	-0.38	-0.67	-0.09	-0.07	-0.21	0.08	-0.07	-0.21	0.07	0.32	-0.07	0.70
Distractibility	0.08	-0.02	0.18	0.04	-0.07	0.14	-0.43	-0.72	-0.15	-0.01	-0.17	0.14	-0.13	-0.27	0.00	-0.07	-0.49	0.36
Persistence	0.09	0.00	0.18	0.00	-0.10	0.10	-0.17	-0.45	0.11	-0.07	-0.23	0.10	-0.08	-0.23	0.07	0.18	-0.25	0.62
Rhythmicity	0.11	0.01	0.21	0.09	-0.01	0.20	-0.31	-0.60	-0.02	0.07	-0.08	0.22	0.02	-0.12	0.17	-0.18	-0.60	0.24
Reactivity	-0.13	-0.23	-0.04	-0.12	-0.23	-0.02	0.32	0.03	0.60	0.02	-0.13	0.16	0.07	-0.08	0.21	0.06	-0.34	0.46

β = standardised regression coefficient. Complete case models included participants with available data on all analysis variables, and were adjusted for all assessed family of origin, sociodemographic, mental health, and behavioural characteristics. Mutual exposure adjustment models were adjusted for all other personality traits. Binary exposure models included the personality trait exposure dichotomised at the 15th %ile, with the risk category representing low levels of the named trait, and were unadjusted.

Supplementary Table 6d. Sensitivity analyses for associations between parent extraversion and perinatal outcomes.

	Mothers									Fathers								
Outcome	Complete case adjusted			Mutual exposure adjustment			Binary exposure			Complete case adjusted			Mutual exposure adjustment			Binary exposure		
Outcome construct	β	95% CI		β	95% CI		β	95% CI		β	95% CI		β	95% CI		β	95% CI	
Periconceptional household income	0.08	-0.04	0.20	0.12	0.01	0.24	-0.28	-0.69	0.14	0.24	0.07	0.40	0.14	0.00	0.28	-0.19	-0.56	0.19
Postnatal household income	0.07	-0.05	0.19	0.12	0.00	0.23	-0.23	-0.65	0.19	0.15	0.00	0.31	0.08	-0.06	0.23	-0.14	-0.51	0.22
Stressful life events	0.09	0.01	0.17	0.19	0.10	0.29	-0.26	-0.62	0.11									
Antenatal social support	0.16	0.06	0.26	0.09	0.00	0.19	-0.56	-0.95	-0.17									
Postnatal social support	0.16	0.06	0.25	0.08	-0.02	0.18	-0.42	-0.79	-0.05									
Partner relationship quality	0.04	-0.04	0.13	-0.02	-0.12	0.09	-0.33	-0.75	0.08									
Antenatal depressive symptoms	-0.18	-0.28	-0.07	0.00	-0.11	0.10	0.37	-0.03	0.77	-0.05	-0.11	0.00	-0.03	-0.08	0.03	0.10	-0.07	0.27
Postnatal depressive symptoms	-0.17	-0.26	-0.08	0.02	-0.07	0.11	0.34	-0.01	0.69									
Parental warmth	0.14	0.04	0.23	0.13	0.03	0.23	-0.06	-0.40	0.28									
Hostile parenting	-0.14	-0.23	-0.04	-0.05	-0.15	0.05	0.19	-0.14	0.51									
Anxious parenting	-0.12	-0.22	-0.02	-0.13	-0.23	-0.03	0.49	0.17	0.81									
Parental self-efficacy	0.23	0.13	0.32	0.13	0.03	0.23	-0.33	-0.65	0.00									
Parent-infant bond	0.21	0.12	0.31	0.11	0.02	0.20	-0.30	-0.61	0.02									
Gestational age at birth	0.04	-0.06	0.13	0.02	-0.08	0.11	-0.19	-0.53	0.16	-0.05	-0.19	0.08	-0.03	-0.17	0.10	0.17	-0.19	0.52
Size for gestational age	-0.05	-0.15	0.06	-0.02	-0.13	0.09	0.11	-0.24	0.47	0.03	-0.10	0.17	0.02	-0.11	0.16	-0.09	-0.45	0.28
Birthweight	-0.03	-0.12	0.07	-0.01	-0.11	0.09	-0.04	-0.36	0.28	-0.04	-0.18	0.10	-0.01	-0.14	0.13	0.02	-0.34	0.39
Approach	0.13	0.04	0.22	0.12	0.02	0.21	-0.11	-0.42	0.20	0.11	-0.01	0.24	0.10	-0.02	0.22	-0.21	-0.54	0.12
Cooperation	0.09	0.00	0.18	0.02	-0.08	0.12	-0.13	-0.46	0.20	0.00	-0.12	0.13	0.02	-0.10	0.14	0.18	-0.14	0.50
Distractibility	0.10	0.00	0.19	0.07	-0.03	0.17	-0.32	-0.65	0.00	0.14	0.01	0.27	0.16	0.04	0.28	-0.20	-0.53	0.13
Persistence	0.08	-0.01	0.17	0.05	-0.04	0.14	-0.02	-0.33	0.29	0.10	-0.04	0.23	0.05	-0.07	0.18	-0.19	-0.54	0.16
Rhythmicity	0.08	-0.02	0.17	0.05	-0.05	0.14	-0.11	-0.43	0.21	0.11	-0.02	0.23	0.05	-0.07	0.17	-0.02	-0.35	0.30
Reactivity	-0.08	-0.17	0.01	0.00	-0.10	0.10	0.29	-0.03	0.61	-0.07	-0.20	0.05	-0.06	-0.18	0.06	0.16	-0.16	0.48

β = standardised regression coefficient. Complete case models included participants with available data on all analysis variables, and were adjusted for all assessed family of origin, sociodemographic, mental health, and behavioural characteristics. Mutual exposure adjustment models were adjusted for all other personality traits. Binary exposure models included the personality trait exposure dichotomised at the 15th %ile, with the risk category representing low levels of the named trait, and were unadjusted.

Supplementary Table 6e. Sensitivity analyses for associations between parent openness and perinatal outcomes.

	Mothers									Fathers								
Outcome	Complete case adjusted			Mutual exposure adjustment			Binary exposure			Complete case adjusted			Mutual exposure adjustment			Binary exposure		
**Outcome construct**	**β**	**95% CI**		**β**	**95% CI**		**β**	**95% CI**		**β**	**95% CI**		**β**	**95% CI**		**β**	**95% CI**	
Periconceptional household income	0.10	-0.03	0.22	0.03	-0.08	0.15	-0.05	-0.46	0.36	0.16	0.01	0.31	0.13	0.01	0.25	-0.37	-0.67	-0.06
Postnatal household income	0.08	-0.05	0.21	-0.01	-0.12	0.10	0.00	-0.42	0.41	0.16	0.02	0.31	0.10	-0.02	0.22	-0.29	-0.60	0.03
Stressful life events	0.01	-0.07	0.10	0.00	-0.09	0.10	0.18	-0.23	0.58									
Antenatal social support	0.10	-0.01	0.20	0.05	-0.05	0.15	-0.18	-0.60	0.25									
Postnatal social support	0.04	-0.06	0.14	-0.01	-0.11	0.08	-0.11	-0.49	0.27									
Partner relationship quality	0.10	0.00	0.19	0.04	-0.07	0.15	-0.02	-0.47	0.44									
Antenatal depressive symptoms	0.03	-0.08	0.15	0.03	-0.08	0.14	0.20	-0.23	0.63	0.01	-0.03	0.06	0.05	0.00	0.10	-0.02	-0.15	0.12
Postnatal depressive symptoms	0.06	-0.03	0.16	0.09	0.00	0.18	-0.05	-0.40	0.30									
Parental warmth	0.13	0.03	0.23	0.11	0.02	0.21	-0.44	-0.78	-0.10									
Hostile parenting	0.06	-0.05	0.16	0.09	-0.01	0.18	-0.29	-0.61	0.03									
Anxious parenting	0.05	-0.05	0.15	0.03	-0.06	0.13	-0.01	-0.35	0.32									
Parental self-efficacy	-0.15	-0.26	-0.05	-0.18	-0.27	-0.09	0.22	-0.10	0.54									
Parent-infant bond	-0.11	-0.21	-0.01	-0.15	-0.24	-0.06	0.11	-0.20	0.43									
Gestational age at birth	0.09	0.00	0.19	0.09	0.00	0.19	-0.09	-0.44	0.27	-0.12	-0.24	0.01	-0.08	-0.19	0.04	0.22	-0.07	0.51
Size for gestational age	-0.01	-0.12	0.10	-0.03	-0.14	0.07	0.04	-0.32	0.41	0.01	-0.11	0.14	-0.02	-0.14	0.10	0.12	-0.19	0.42
Birthweight	0.06	-0.04	0.16	0.03	-0.06	0.12	-0.02	-0.34	0.31	-0.09	-0.22	0.04	-0.06	-0.18	0.06	0.22	-0.07	0.51
Approach	0.05	-0.05	0.15	0.02	-0.07	0.11	0.03	-0.28	0.35	0.14	0.02	0.25	0.08	-0.02	0.19	-0.17	-0.44	0.10
Cooperation	-0.04	-0.14	0.05	-0.06	-0.16	0.03	-0.01	-0.34	0.32	0.00	-0.12	0.11	-0.02	-0.12	0.09	0.11	-0.16	0.38
Distractibility	0.07	-0.03	0.17	0.03	-0.06	0.12	-0.07	-0.39	0.26	0.04	-0.07	0.16	0.07	-0.03	0.17	-0.09	-0.36	0.18
Persistence	0.06	-0.03	0.15	0.06	-0.03	0.15	-0.39	-0.70	-0.09	0.08	-0.05	0.21	0.10	-0.01	0.22	-0.23	-0.52	0.05
Rhythmicity	-0.06	-0.16	0.04	-0.06	-0.16	0.03	0.01	-0.31	0.34	0.11	0.00	0.23	0.07	-0.04	0.17	-0.07	-0.33	0.20
Reactivity	-0.02	-0.11	0.08	0.00	-0.09	0.10	0.14	-0.18	0.46	-0.03	-0.14	0.08	-0.01	-0.12	0.09	-0.09	-0.35	0.18

β = standardised regression coefficient. Complete case models included participants with available data on all analysis variables, and were adjusted for all assessed family of origin, sociodemographic, mental health, and behavioural characteristics. Mutual exposure adjustment models were adjusted for all other personality traits. Binary exposure models included the personality trait exposure dichotomised at the 15th %ile, with the risk category representing low levels of the named trait, and were unadjusted.

Supplementary Figure 1. Sampling and ascertainment of the Victorian Intergenerational Health Cohort, 2006-2014.

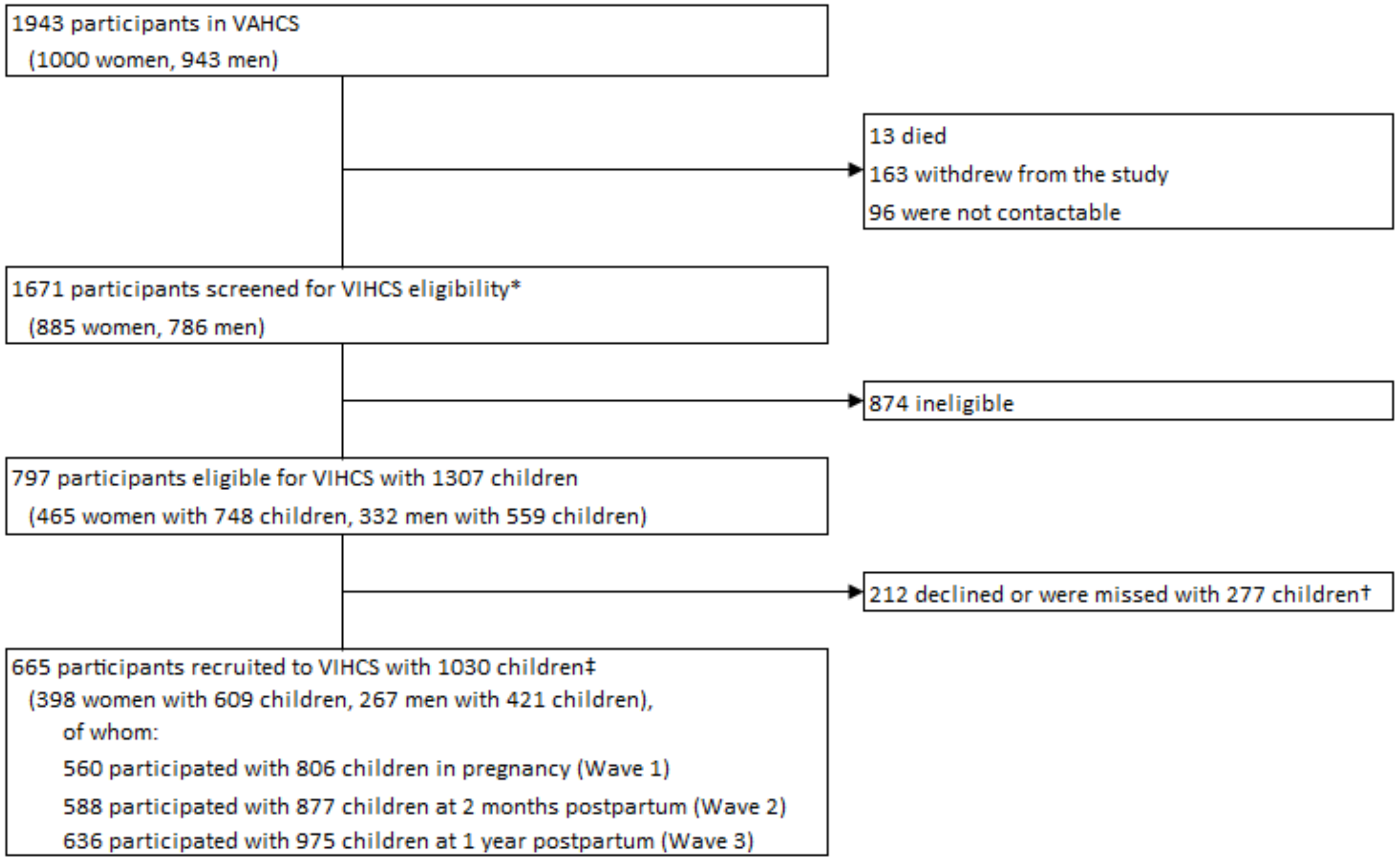

* Eligibility for study entry at VIHCS wave 1 defined as a viable pregnancy in trimester three of pregnancy during VIHCS screening (1 September 2006 to 30 June 2013); eligibility for study entry at VIHCS wave 2/3 defined as a live birth during VIHCS screening.

† Of the 212 VAHCS participants who didn't participate for one or more eligible VIHCS children, 132 were completely excluded and the remaining 80 were recruited to participate in the study with at least one other child.

‡ The cohort remained open during Waves 1-3, so that parents could enter the study at Wave 2 or 3 if they had missed earlier wave/s. Many parents participated with more than one child born during the VIHCS recruitment phase.
